# Maternal High-Fat Diet Disturbs the DNA Methylation Profile in the Brown Adipose Tissue of Offspring Mice

**DOI:** 10.3389/fendo.2021.705827

**Published:** 2021-10-08

**Authors:** Qian Zhang, Xinhua Xiao, Jia Zheng, Ming Li, Miao Yu, Fan Ping, Tong Wang, Xiaojing Wang

**Affiliations:** Key Laboratory of Endocrinology, Ministry of Health, Department of Endocrinology, Peking Union Medical College Hospital, Peking Union Medical College, Chinese Academy of Medical Sciences, Beijing, China

**Keywords:** maternal exposure, obesity, developmental programming, DNA methylation, high fat diet

## Abstract

The prevalence of obesity has become a threatening global public health issue. The consequence of obesity is abnormal energy metabolism. Unlike white adipose tissue (WAT), brown adipose tissue (BAT) has a unique role in nonshivering thermogenesis. Lipids and glucose are consumed to maintain energy and metabolic homeostasis in BAT. Recently, accumulating evidence has indicated that exposure to excess maternal energy intake affects energy metabolism in offspring throughout their life. However, whether excess intrauterine energy intake influences BAT metabolism in adulthood is not clear. In this study, mouse dams were exposed to excess energy intake by feeding a high-fat diet (HFD) before and during pregnancy and lactation. The histology of BAT was assessed by hematoxylin and eosin staining. The genome-wide methylation profile of BAT was determined by a DNA methylation array, and specific site DNA methylation was quantitatively analyzed by methylated DNA immunoprecipitation (MeDIP) qPCR. We found that intrauterine exposure to a high-energy diet resulted in blood lipid panel disorders and impaired the BAT structure. Higher methylation levels of genes involved in thermogenesis and fatty acid oxidation (FAO) in BAT, such as *Acaa2*, *Acsl1*, and *Cox7a1*, were found in 16-week-old offspring from mothers fed with HFD. Furthermore, the expression of *Acaa2*, *Acsl1*, and *Cox7a1* was down-regulated by intrauterine exposure to excess energy intake. In summary, our results reveal that excess maternal energy leads to a long-term disorder of BAT in offspring that involves the activation of DNA methylation of BAT-specific genes involved in fatty acid oxidation and thermogenesis.

## Introduction

Obesity remains a significant global public health issue (1). Currently, the obesity and overweight occur not only in developed and developing countries but also in undeveloped regions. Obesity is involved in multiple diseases, such as type 2 diabetes, cardiovascular disease, nonalcoholic steatohepatitis, and even cancer (2–5). Obesity is a multi-factorial disease. In addition to genetic factors, environmental changes also contribute to the incidence of obesity, especially during critical developmental periods, such as gestation and lactation (6). Epidemiological data indicates that a maternal high-fat diet is one of the key risk factors for childhood obesity (7, 8). Obesity during childhood is strongly associated with obesity and other unhealthy outcomes in adulthood (9). Moreover, animal research has demonstrated that exposure to excess energy intake during early life stages, such as the fetal and infancy periods, induces obesity and dysfunction in adipose tissue in adulthood (10–13). Increasing evidence supports the developmental origins of a health and diseases (DOHaD) concept; that is, exposure during an early life period is the origin of the development of health and diseases in adulthood (14). This phenomenon is also explained as metabolic programming, which is defined as adverse exposure during gestation and lactation that programs the health and illnesses in adulthood (15, 16). Epigenetic mechanisms, including DNA methylation, chromatin remodel, and noncoding RNAs (17, 18), can influence gene expression chiefly at the level of transcription (19) and play a critical role in metabolic programming (20). Among these epigenetic mechanisms, DNA methylation, which takes place at the 5’ position of cytosine in CpG islands at specific locations in genome, has been proven to be a key mechanism in the progression of obesity and lipid metabolism (20). DNA methylation, which occurs in CpG-rich regions known as CpG islands in the genome, can result in gene expression alteration (21).

In mammals, white adipose tissue (WAT) is at high ratio in the body and is well studied (22). As a primary site of excess energy storage, WAT hypertrophy is closely related to obesity. Another type of adipose tissue is brown adipose tissue (BAT). The main function of BAT is nonshivering thermogenesis. In BAT, fatty acids and glucose are utilized as fuel to produce heat under stimuli (22, 23). In the past, the BAT was considered only to exist rodent animals and newborn babies. However, recent studies have confirmed that active BAT depots also exist in adult humans (24). Recently, scientists discovered the important role of BAT in adults and its potential strategy for obesity treatment (25).

However, the majority of studies focus on the effect of maternal HFD on the morphological and functional disorders of WAT in offspring. These studies reveal that maternal obesity is closely related to increased WAT, and glucose and lipid metabolism disorders during the adult life of offspring (26–30). Several studies have proven that maternal obesity also disturbs the thermogenesis function of BAT in offspring, resulting in obesity in adulthood (31–33).

In this study, we hypothesized that maternal HFD exposure affects offspring BAT morphology, biological function, and DNA methylation modification, which boosts the progression of obesity. Therefore, we focused on whole-genomic methylation in the BAT from offspring exposed to a maternal HFD, as evaluated by a DNA methylation array.

## Materials and Methods

### Animal Treatments and Diets

**Figure 1:**
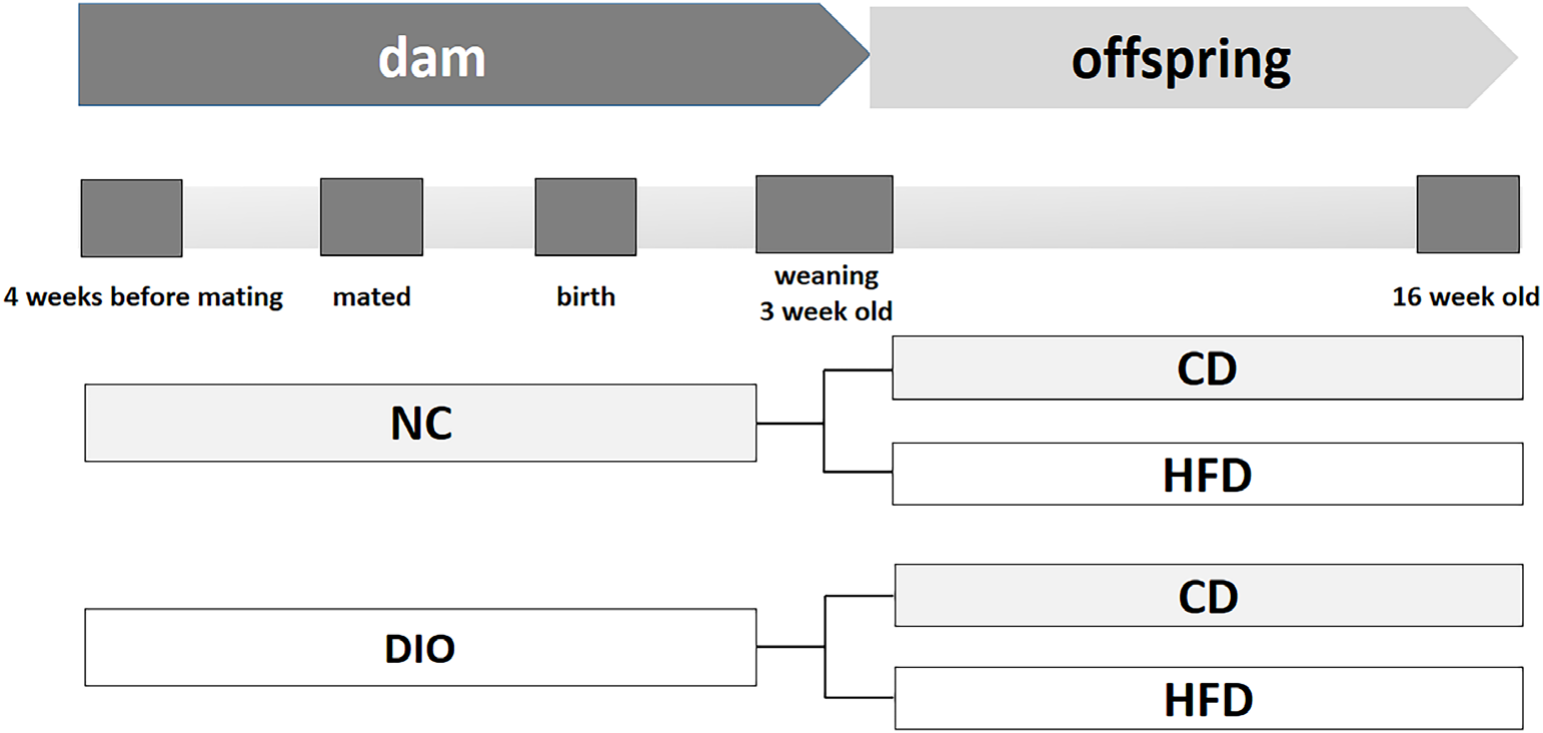
Schema of animal experiment protocol. NC, normal control; DIO, diet induced obesity; CD, control diet; HFD, high-fat diet.

This work was designed according to the NIH guidelines for the use of laboratory animals (NIH Publications No.86-23, revised 1996). All experimental protocols were approved by the Animal Care Committee of Peking Union Medical Hospital (Permit Number: XHDW-2015-0051).Thirty-two female C57BL/6J mice (five weeks old) were purchased from the Institute of Laboratory Animal Science, Chinese Academy of Medical Sciences and Peking Union Medical College (Beijing, China). Sixteen females were randomly assigned to consume a normal control rodent diet (NC, 16% kcal fat, 64% kcal carbohydrate, 20% kcal protein, D11112201, Research Diets, New Brunswick, NJ). Another sixteen mice were fed a high-fat diet (HFD, 45% kcal fat, 35% kcal carbohydrate, 20% kcal protein, D12451, Research Diets, New Brunswick, NJ) to induce obesity. After a 4-week diet scheme, female mice were mated with male mice fed with a normal diet. Female mouse diet protocols were continued during the following gestation and lactation periods. Thus, two different diet-exposed dams were obtained: normal control (NC) mice fed a normal diet and diet-induced obesity (DIO) mice fed a HFD. After birth, litters were reduced to six pups (3 males and 3 females) per mother to avoid competition for milk. After weaning, only male offspring (one male pup from each litter was randomly assigned to the experimental groups) were randomly divided into the following groups to receive a postweaning control diet (CD) or a HFD (n = 8 each group) for 13 weeks. Other surviving mice were kept feeding for other study. Thus, four subgroups were obtained, NC-CD, NC-HFD, DIO-CD, and DIO-HFD, to research the interaction between the maternal and offspring diets on the progression of obesity (**Figure 1**). The female offspring were not included in this study, because of confounding factors related to their hormone profile and estrus cycle. Food consumption and body weight were monitored. Offspring were sacrificed at 16 weeks old.

### Sample and Tissue Collection

After an overdose injection of pentobarbital sodium (150 mg/kg), mice were sacrificed. Other surviving mice were kept feeding. Then, blood was collected by cardiac puncture. Interscapular BAT was separated and weighed. Part of the BAT was frozen in liquid nitrogen and stored at -80°C for future molecular experiments. The other part of the BAT was fixed in 10% formalin for histological analyses.

### Metabolic Analyses of Circulating Factors

Serum was collected from mice that were fasted overnight. Total cholesterol (TC) and triglyceride (TG) levels were assayed by enzyme end-point assays with a commercial kit (Roche Diagnostics, GmbH, Mannheim, Germany) according to the manufacturer’s instructions.

### Hematoxylin and Eosin (H&E) Staining

The BAT was fixed in 4% paraformaldehyde. Then, tissues were embedded in paraffin. Five micrometer sections were cut by a microtome (Nickon, Tokyo, Japan). Finally, the section was stained with H&E. Photographs (magnification 400 x) were taken using a Nikon microscope (Nikon, Tokyo, Japan). Lipid area was measured by Image-Pro Plus version 7.0 (Media Cybernetics Inc., Rockville, MD). Lipid droplets in the histopathological image were defined as circular unstained objects, as descripted in previous reference (34).

### DNA Preparation and DNA Methylation Microarray

To determine the effect of maternal DIO on DNA methylation in offspring BAT, genomic DNA was extracted from BAT using a DNeasy Blood & Tissue Kit (Qiagen, Valencia, CA). Isolated genomic DNA from the DIO-CD group and NC-CD group (n = 3 for each group) was fragmented to 200 to 1,000 bp by sonication. Through reaction with magnetic beads coupled with mouse monoclonal antibodies against 5-methylcytidine (Diagenode, Liege, Belgium), the methylated DNA was immunoprecipitated. The total input DNA was labeled with Cy3-labeled random 9-mers, while the immunoprecipitated DNA was labelled with Cy5-labeled random 9-mers. Then, labelled DNA was hybridized to an Arraystar Mouse RefSeq Promoter Array (Arraystar Inc., Rockville, MD). This array covers 22,327 well-characterized RefSeq promoter regions with ∼180,000 probes. The promoter regions were defined as ∼-1,300 to +500 bp of the transcription start sites (TSSs). The array was scanned by an Agilent Scanner G2505C (Agilent Technologies, Waldbronn, Germany).

### Data Normalization and Analysis

Raw data were generated and normalized to log 2 ratio data and then analyzed to identify enriched peaks by NimbleScan Software v2.5 (Roche NimbleGen Inc., Madison, WI). To calculate the difference in enriched peaks between DIO-CD and NC-CD offspring BAT, the average log2 ratios and M’ were calculated for each probe, according to the following formula,


$$M'=average\left[\log2^{MeDIP\left(DIO-CD\right)/Input\left(DIO-CD\right)}\right]-average\left[log2^{MeDIP\left(NC-CD\right)/Input\left(NC-CD\right)}\right]$$

The NimbleScan sliding-window peak-finding algorithm was used to find the differentially enriched peaks (DEPs). DEPs were defined as follows, (i) at least one of the two groups had a median value of log_2_(MeDIP/Input) ≥ 0.3 and a median value of M’ > 0 within the peak; and (ii) at least half of the probes in a peak had a median value of the coefficient of variability (CV) ≤ 0.8 for both groups. Then, PeakScores were calculated to identify differentially methylated regions. The DEP was defined as follows: PeakScore ≥ 2 and *P* ≤ 0.01. The CpG region was categorized according to CpG island density, including high CpG promoters/regions (HCPs), intermediate CpG promoters/regions (ICPs), and low CpG promoters/regions (LCPs) (35). All differentially methylated genes (DMGs) were clustered to Gene Ontology (GO) and Kyoto Encyclopedia of Genes and Genomes (KEGG) pathway terms by using DAVID Bioinformatics Resources version 6.7 (http://david.abcc.ncifcrf.gov/) (36). Significant GO functional clusters and KEGG pathways were defined as the follows, at least 2 DMGs in each GO and/or KEGG pathway term, a Benjamini–Hochberg FDR *P* < 0.05 (KEGG) or 0.01 (GO) and a fold enrichment ≥ 1.50 (KEGG) or 1.10 (GO) within each cluster.

### Methylated DNA Immunoprecipitation (MeDIP) qPCR

To detect the methylation status of specific genes, MeDIP qPCR was used. Genomic DNA was fragmented by ultrasonication. DNA fragments of 100∼500 bp on average were obtained. Similar to DNA methylation microarray, denatured DNA containing RNase A was incubated with magnetic beads coupled with mouse monoclonal antibodies against 5-methylcytidine antibody (Zymo Research, Irvine, CA) or mouse anti-IgG overnight at 4°C. Then, the mixture was incubated with protein G magnetic beads at 4°C for 1 h, washed in digestion buffer at 65°C for 3 h, and finally quantified by quantitative PCR (qPCR). CpG island analyses and MeDIP qPCR primer designs were based on the genome-wide epigenetic database of the National Center for Biotechnology Information and MethPrimer 1.0 (37). MeDIP qPCR primers are listed in **Table 1**.

**Table 1:** Primers for MeDIP qPCR.

Genes	Genbank ID	Forward primer	Reverse primer	Production size (bp)
Acaa2	NM_177470	TGGCTAGGCTCCTGACCTTT	CTCTGCTCCAGACCACTTCG	108
Acsl1	NM_001302163	GGAAGAGCTAAAGGGCACCT	GGTAGGGCAAAGGGTAAATC	85
Cox7a1	NM_009944	CCCAGATTATCAGCAGGGTA	TGACAGTGGCTCAGGGACTT	66

Acaa2, acetyl-coenzyme A acyltransferase 2; Acsl1, acyl-CoA synthetase long-chain family member 1; Cox7a1,cytochrome c oxidase subunit 7A1.

### RNA Isolation and qPCR Analysis

Total RNA was extracted by using TRIzol reagent (Invitrogen, Carlsbad, CA), according to the manufacturer’s instructions. The expression of acetyl-coenzyme A acyltransferase 2 (*Acaa2*), acyl-CoA synthetase long-chain family member 1 (*Acsl1*) and cytochrome c oxidase subunit 7A1, mitochondrial (*Cox7a1*) was measured using an ABI Prism 7900 system (Applied Biosystems, Foster City, CA) by the comparative Ct method (2^-△△Ct^). The relative RNA expression levels were standardized to *Gadph*. The primer sequences are listed in **Table 2**.

**Table 2:** Primers for qPCR.

Genes	Genbank ID	Forward primer	Reverse primer	Production size (bp)
Acaa2	NM_177470	AGACCATGCAAGTGGACGAG	CCAGGGGCGTGAAGTTATGT	186
Acsl1	NM_001302163	GTGTAGGACTCGGCATGTGA	TCAGAAAAGGGCAGTGAGGC	112
Cox7a1	NM_009944	AAAGTGCTGCACGTCCTTG	CCCGCCTTTCAAGTGTACTG	198

Acaa2, acetyl-coenzyme A acyltransferase 2; Acsl1, acyl-CoA synthetase long-chain family member 1; Cox7a1,cytochrome c oxidase subunit 7A1.

### Statistical Analysis

The research data are shown as the mean ± SEM. Differences between the two dam groups were analyzed using Student’s *t* test. Differences among the four offspring groups were analyzed using two-way ANOVA to analyze the maternal diet effect, offspring diet effect and their interaction. Prism Software version 7 (GraphPad Software Inc., San Diego, CA) was used to perform statistical analyses. Statistical significance was defined as *P* < 0.05.

## Results

### The Effect of Maternal HFD on Food Intake and Body Weight

**Figure 2:**
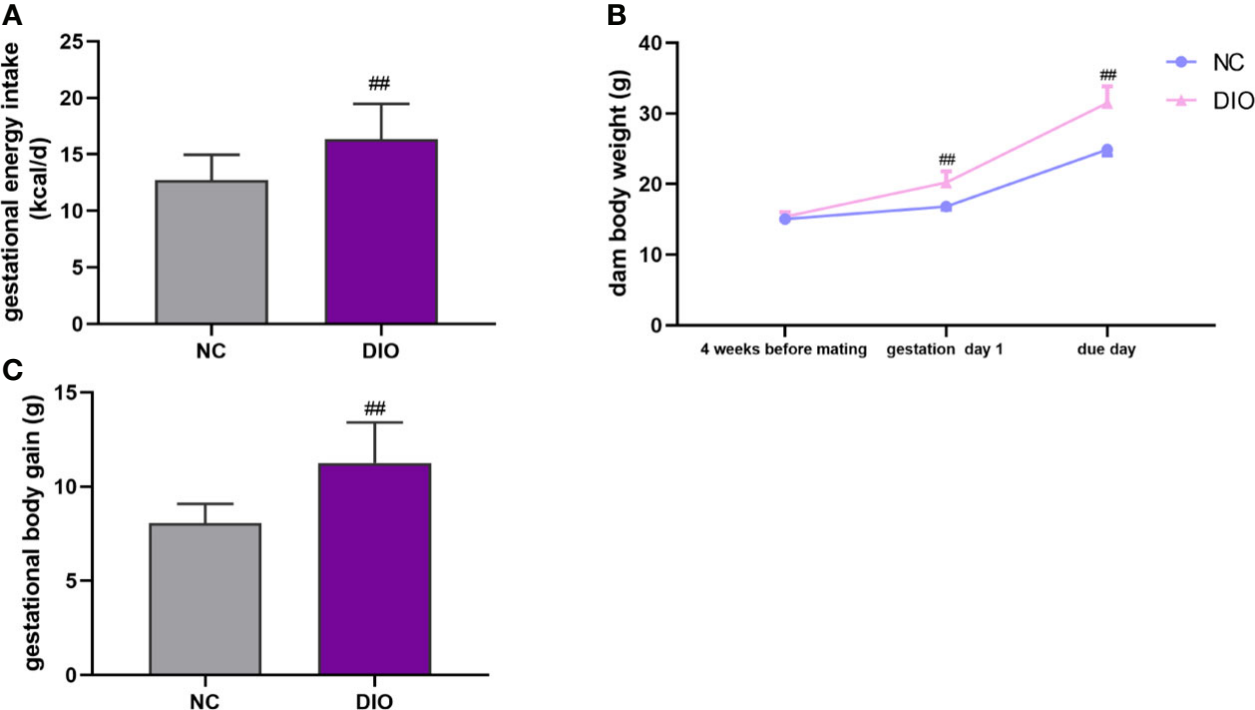
Maternal characteristics. Maternal **(A)** energy intake during pregnancy, **(B)** body weight and **(C)** body weight gain during pregnancy. Values represent the mean ± SEM, n = 16 each group. ^##^ *P* < 0.01 DIO *vs*. NC group. NC, normal control; DIO, diet-induced obesity.

During pregnancy, DIO dams consumed more caloric energy than NC dams (*P* < 0.01, **Figure 2A**). After 4-week HFD, DIO mice model was successfully established (**Figure 2B**). On the deliver day, the weight of DIO group was higher than that of NC group (*P* < 0.01, **Figure 2B**). And DIO females had a 1.40-fold increase in gestational body weight gain compared with NC females (*P* < 0.01, **Figure 2C**).

### The Effect of Maternal HFD on Body Weight and BAT Weight in Male Offspring

**Figure 3:**
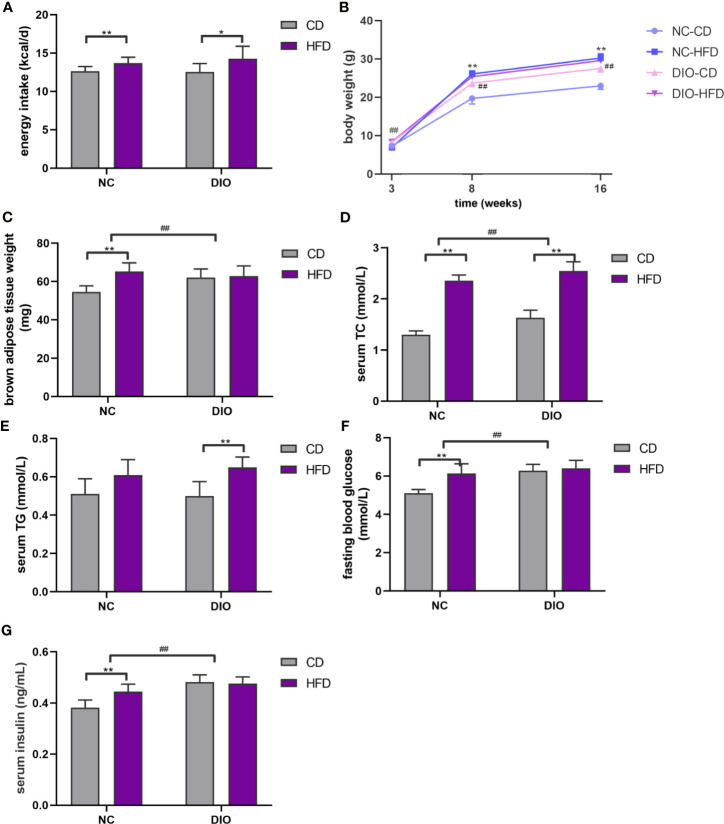
Offspring characteristics. **(A)** Energy intake, **(B)** body weight, **(C)** brown adipose tissue weight (BAT), **(D)** serum total cholesterol (TC), **(E)** serum triglyceride (TG), **(F)** fasting blood glucose (FBG), **(G)** serum insulin. Values represent the mean ± SEM, n = 8 each group. *P* values represent significance in the main effect for each source of variation (diet or prenatal exposure) as calculated by two-way ANOVA: *P < 0.05, **P < 0.01 offspring diet effect, ^##^ *P* < 0.01 maternal diet effect. NC, normal control; DIO, diet-induced obesity; CD, control diet; HFD, high-fat diet.

There were no differences in litter sizes (7.00 ± 0.73 *vs*. 7.38 ± 0.62), male: female ratio (0.47 ± 0.06 *vs* 0.50 ± 0.09), and birth weight (1.33 ± 0.09 g *vs* 1.27 ± 0.06 g) between offspring from DIO and NC dams (*P* > 0.05). Offspring fed with HFD had more energy intake at 16 weeks old (diet exposure *P* < 0.05 or 0.01, **Figure 3A**). In all groups of offspring, the HFD resulted in an increase in the body weight of male offspring at 8 and 16 weeks old (diet exposure *P* < 0.01, **Figure 3B**). Although there is no significant difference of energy intake between DIO-CD and NC-CD group. Maternal DIO induced a significant increase in body weight (prenatal exposure *P* < 0.01, **Figure 3B**). Although BAT weight was elevated in NC-HFD offspring compared to NC-CD offspring (diet exposure *P* < 0.01, **Figure 3B**), BAT weight in DIO-HFD offspring was similar to that in DIO-CD offspring. Notably, BAT weight in DIO-CD offspring was higher than that in NC-CD offspring (prenatal exposure *P* < 0.01, **Figure 3C**).

### The Effect of Maternal HFD on the Serum Lipid Profile in Male Offspring

Serum TC levels were 1.82-fold higher in the NC-HFD offspring than in the NC-CD offspring (diet exposure *P* < 0.01, **Figure 3D**). Furthermore, DIO-HFD offspring exhibited more hyperlipidemia than NC-CD offspring (1.96-fold, diet exposure *P* < 0.01, prenatal exposure *P* < 0.01, **Figure 3D**). However, elevated serum TG levels were observed only in the DIO-HFD group, compared with those in the DIO-CD group (diet exposure *P* < 0.01, **Figure 3E**).

### The Effect of Maternal HFD on the Fasting Blood Glucose and Serum Insulin in Male Offspring

Mice in NC-HFD had higher fasting blood glucose (FBG) and serum insulin than those of NC-CD group (diet exposure *P* < 0.01, **Figures 3F, G**). And FBG and serum insulin in DIO-CD offspring was also higher than that in NC-CD group (prenatal exposure *P* < 0.01, **Figures 3F, G**).

### The Effect of Maternal HFD on the Morphology of BAT in Male Offspring

**Figure 4:**
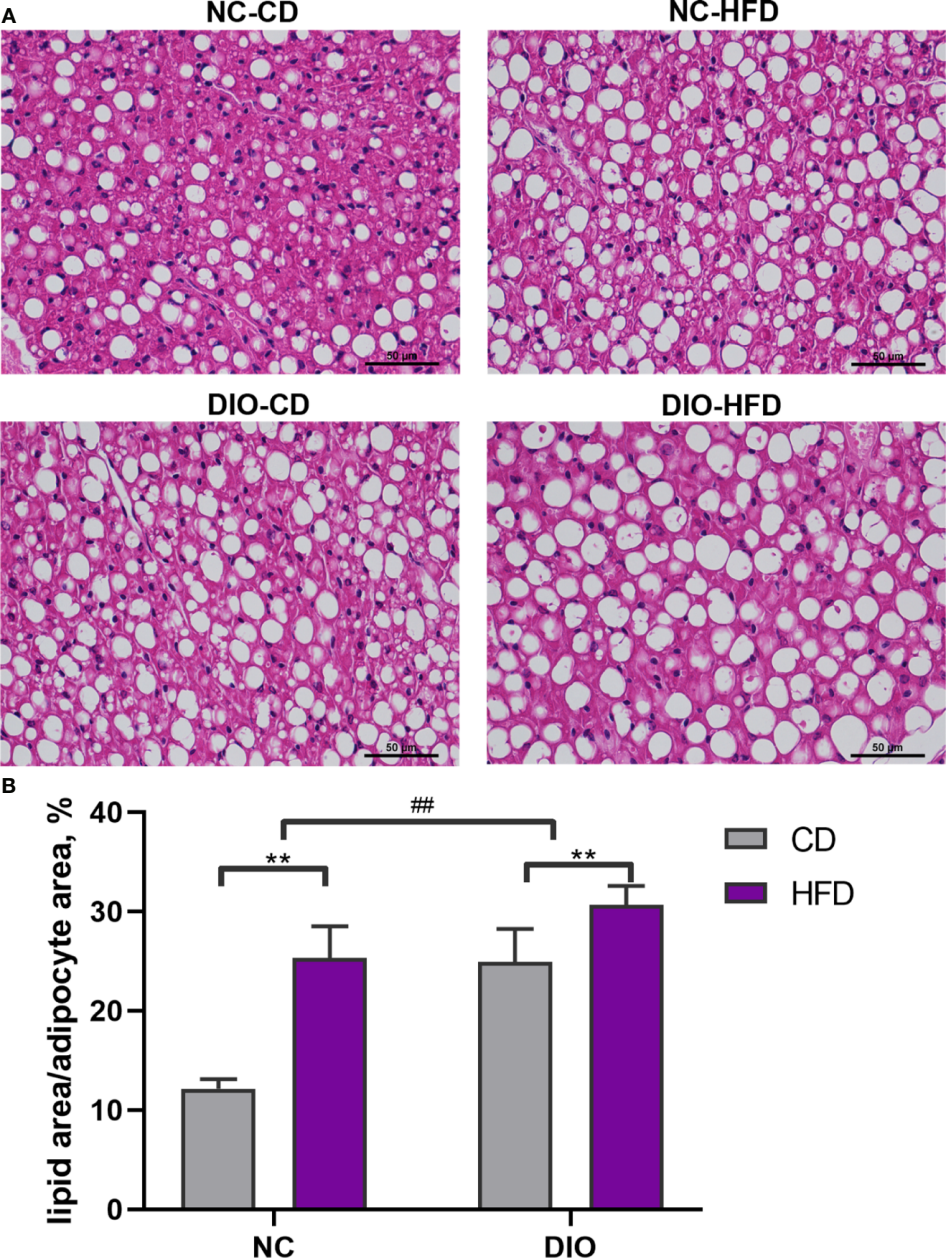
The effect of maternal HFD disrupts the morphology of brown adipose tissue (BAT) in male offspring. **(A)** H&E staining (magnification 400 x), **(B)** lipid area analysis (lipid area/total adipocyte area, %) of BAT (n = 8 for each group). Values represent the mean ± SEM, n = 8 each group. *P* values represent significance in the main effect for each source of variation (diet or prenatal exposure) as calculated by two-way ANOVA: **P < 0.01 offspring diet effect, ^##^ *P* < 0.01 maternal diet effect. NC, normal control; DIO, diet-induced obesity; CD, control diet; HFD, high-fat diet.

H&E stained slides showed that the NC-HFD, DIO-CD, and DIO-HFD groups had large brown adipocytes and white-like adipocytes with unilocular lipid droplets (**Figure 4A**). The fat area of the BAT in both HFD groups was 2.09-fold and 1.23-fold higher than that in the CD group (diet exposure *P* < 0.01, **Figure 4B**). DIO-HFD offspring had a 2.52-fold increase in the fat area of BAT compared with NC-CD offspring (diet exposure *P* < 0.01, prenatal exposure *P* < 0.01, **Figure 4B**).

### The Effect of Maternal HFD on Genome Methylation in BAT of Male Offspring

**Figure 5:**
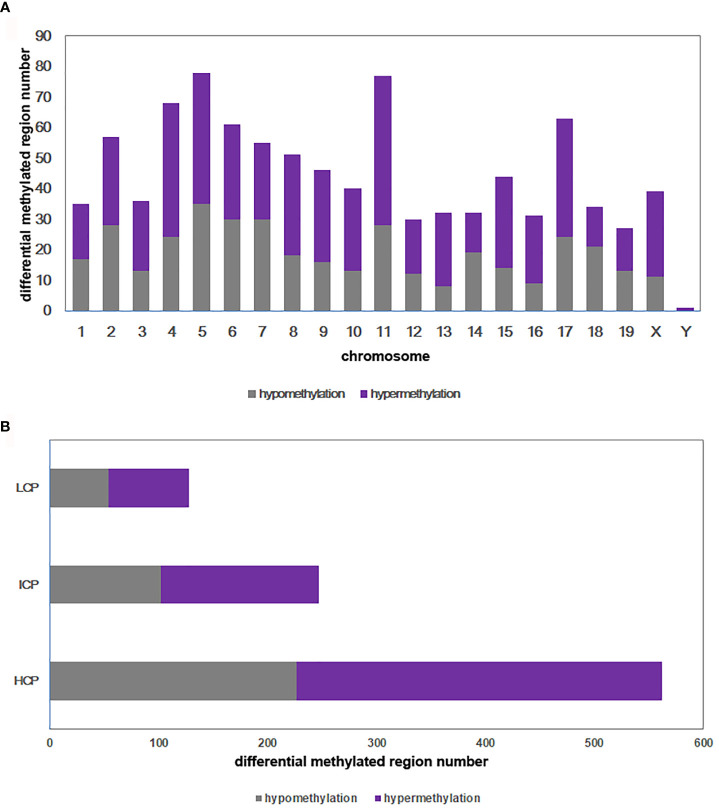
Differential methylated regions between DIO-CD group and NC-CD group. **(A)** Chromosomal distribution of differentially methylated regions. **(B)** CpG density of differentially methylated regions. Classification of all regions with high (HCP), intermediated (ICP), and low (LCP) CpG content.

DNA methylation array data were uploaded to NCBI’s Gene Expression Omnibus repository (GEO) under the series accession number GSE173218 (https://www.ncbi.nlm.nih.gov/geo/query/acc.cgi?acc=GSE173218). An overall comparison of the DNA methylation of BAT in the DIO-CD and NC-CD groups was performed. In total, 937 regions on 21 chromosomes were differentially methylated in the DIO-CD group compared with the NC-CD group, particularly on chromosomes 4, 5, 6, 11, and 17 (**Figure 5A**). Of these differentially methylated regions (DMRs), 554 were hypermethylated (59.12%) and 383 were hypomethylated (40.88%) of the DMRs (**Supplementary Table 1**). Five hundred sixty-two DMRs (59.98%) were located in HCP, 247 DMRs (26.36%) were located in ICP, and 128 DMRs (13.66%) were located in LCP (**Figure 5B**).

### The Effect of Maternal HFD on BAT Methylated Gene Functional Clusters in Male Offspring

**Figure 6:**
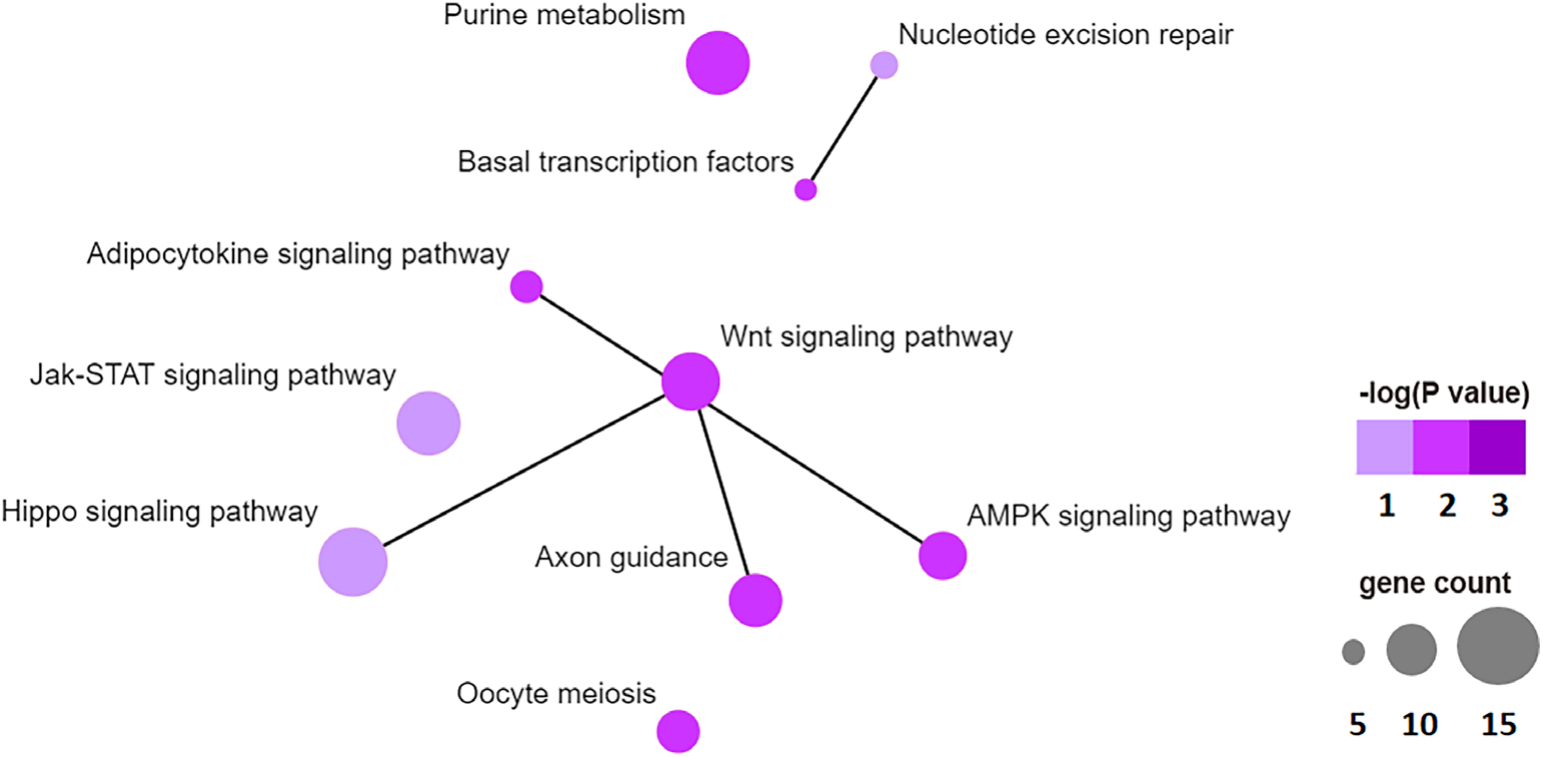
Top 10 significant enriched KEGG pathway network, like hippo signaling pathway, Jak-STAT signaling pathway, nucleotide excision repair, WNT signaling pathway, axon guidance, adipocytokine signaling pathway, purine metabolism, basal transcription factors, AMPK signaling pathway, oocyte meiosis.

To examine the biological function of differentially methylated genes in BAT from offspring exposed to maternal HFD, GO and KEGG enrichment analyses were performed. **Table 3** presents the top five prevalent GO terms for differentially methylated genes. The top five significant biological process (BP) GO terms were transcription, negative regulation of cell proliferation, protein ubiquitination, negative regulation of transcription from the RNA polymerase II promoter, and regulation of transcription. **Table 4** presents the enriched KEGG pathways for differentially methylated genes. The relationship of this enriched KEGG pathways was shown in **Figure 6**. The pathway connections in this figure were based on the KEGG database. The differentially methylated genes were most significantly enriched in the Hippo signaling pathway, Jak-Stat signaling pathway, nucleotide excision repair, Wnt signaling pathway, axon guidance, adipocytokine signaling pathway, purine metabolism, basal transcription factors, AMPK signaling pathway, and oocyte meiosis.

**Table 3:** The enriched GO terms with differentially methylated genes in DIO-CD offspring *vs* NC-CD offspring (*P* < 0.01).

Catalog	Term ID	Term name	Gene Count	Fold Enrichment	*P* value
BP	GO:0006351	transcription, DNA-templated	107	1.466	4.48 x 10^-5^
BP	GO:0008285	negative regulation of cell proliferation	28	1.884	0.00211
BP	GO:0016567	protein ubiquitination	26	1.855	0.00380
BP	GO:0000122	negative regulation of transcription from RNA polymerase II promoter	44	1.559	0.00399
BP	GO:0006355	regulation of transcription, DNA-templated	113	1.281	0.00406
CC	GO:0005634	nucleus	294	1.307	3.15 x 10^-8^
CC	GO:0005737	cytoplasm	312	1.259	4.07 x 10^-7^
CC	GO:0031594	neuromuscular junction	10	4.180	5.87 x 10^-4^
CC	GO:0016020	membrane	304	1.162	6.50 x 10^-4^
CC	GO:0005654	nucleoplasm	11	3.270	0.00192
MF	GO:0005515	protein binding	209	1.318	4.77 x 10^-6^
MF	GO:0003677	DNA binding	102	1.425	2.03 x 10-^4^
MF	GO:0019904	protein domain specific binding	24	2.143	8.98 x 10^-4^
MF	GO:0044822	poly(A) RNA binding	65	1.507	9.41 x 10^-4^
MF	GO:0008270	zinc ion binding	63	1.512	0.00104

BP, biological processes; CC, cellular components; MF, molecular function.

**Table 4:** The enriched KEGG pathway with differentially methylated genes in DIO-CD offspring *vs* NC-CD offspring (*P* < 0.05).

Term ID	Term name	Gene Count	Fold Enrichment	*P* value	Genes
mmu04390	Hippo signaling pathway	16	2.698	8.02 x 10^-4^	CRB2, WWC1, FZD6, BMP8A, FZD8, FZD10, CSNK1E, LIMD1, GDF7, SOX2, WNT6, PPP2R1B, PPP2R2B, CDH1, CTNNA1, TEAD3
mmu04630	Jak-STAT signaling pathway	14	2.459	0.0044	OSM, PIK3CD, OSMR, PIAS2, IL22RA1, SOCS3, IFNE, LEPR, IFNK, EP300, JAK2, IL13RA1, SOCS5, CRLF2
mmu03420	Nucleotide excision repair	7	4.052	0.0069	DDB1, POLD3, ERCC4, CCNH, GTF2H1, GTF2H5, RBX1
mmu04310	Wnt signaling pathway	13	2.348	0.0091	CAMK2D, ROCK2, CTBP1, FZD6, FZD8, FZD10, CSNK1E, RBX1, PPP3CA, WNT6, PPP3CB, CSNK2B, EP300
mmu04360	Axon guidance	12	2.369	0.0121	SEMA6B, EFNB2, EPHA4, PPP3CA, PPP3CB, CDK5, ROCK2, SLIT2, PLXNC1, PTK2, ROBO1, EPHB3
mmu04920	Adipocytokine signaling pathway	8	2.830	0.0220	SOCS3, STK11, G6PC, ACSL1, TRADD, LEPR, JAK2, PCK2
mmu00230	Purine metabolism	14	1.992	0.0231	GUCY1A3, RRM1, PDE4C, ENTPD8, NME4, HPRT, HDDC3, POLD3, NT5E, ADCY9, POLR3D, PDE4B, POLR1D, POLR3G
mmu03022	Basal transcription factors	6	3.473	0.0277	GTF2A1L, TBP, CCNH, GTF2H1, TAF9B, GTF2H5
mmu04152	AMPK signaling pathway	11	2.189	0.0282	CCNA1, STK11, G6PC, PPP2R1B, RAB14, CAB39L, PPP2R2B, PPP2R5B, LEPR, PIK3CD, PCK2
mmu04114	Oocyte meiosis	10	2.274	0.0310	PPP3CA, PPP3CB, CAMK2D, ADCY9, PPP2R1B, STAG3, PPP2R5B, ANAPC5, CPEB2, RBX1

### The Effect of Maternal HFD on BAT-Specific Gene Methylation in Male Offspring by Using MeDIP qPCR

**Figure 7:**
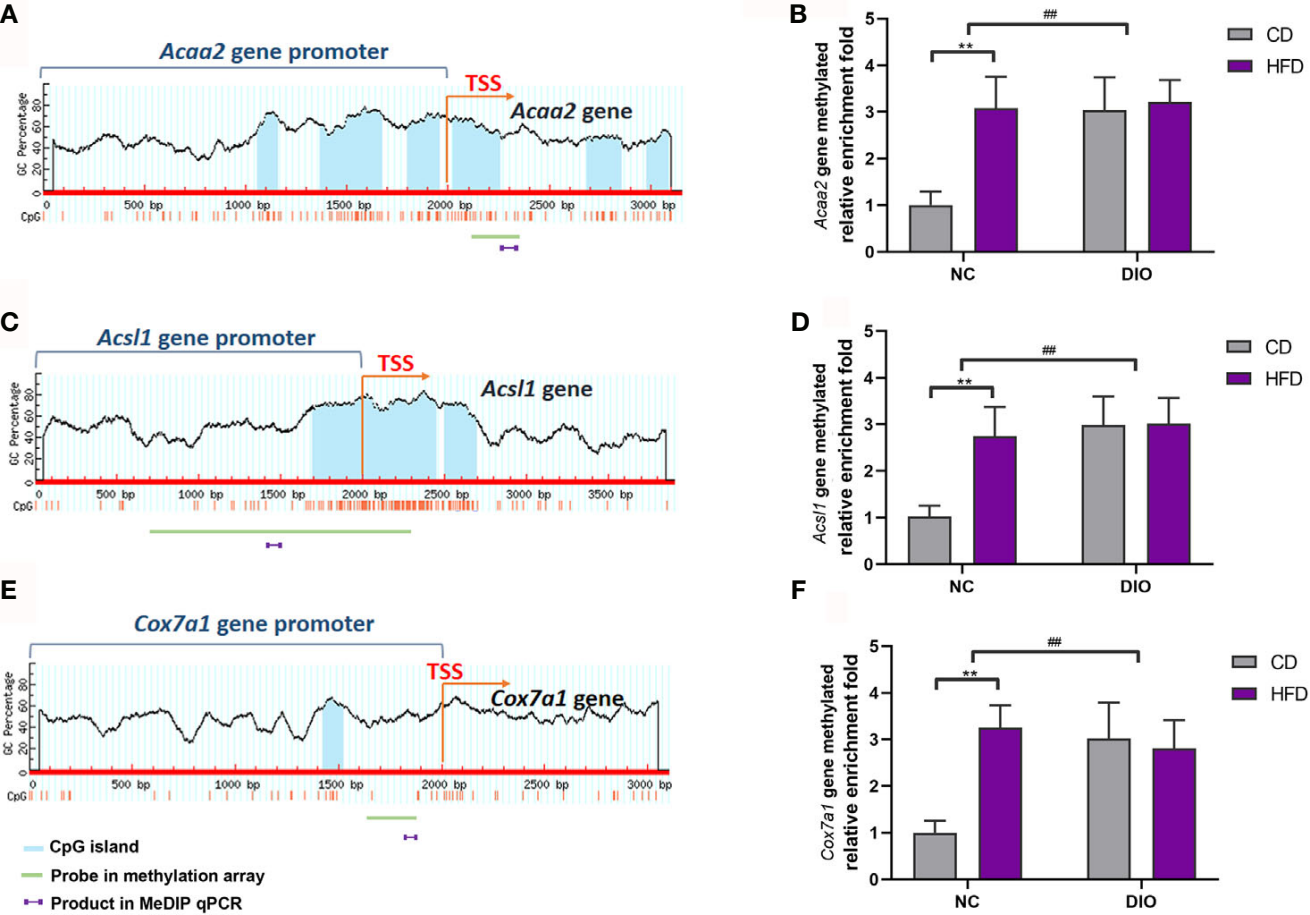
The effect of maternal HFD activated the gene methylation of *Acaa2*, *Acsl1*, and *Cox7a1* promoter in male offspring BAT. Diagram showed the CpG island region in promoters and transcriptional starting site (TSS) shores of **(A)** *Acaa2*, **(C)** *Acsl1*, and **(E)** *Cox7a1*. The methylation changes of **(B)** *Acaa2*, **(D)** *Acsl1*, **(F)** *Cox7a1* quantified by MeDIP-qPCR in BAT. Values represent the mean ± SEM, n = 8 each group. P values represent significance in the main effect for each source of variation (diet or prenatal exposure) as calculated by two-way ANOVA: **P < 0.01 offspring diet effect, ^##^ *P* < 0.01 maternal diet effect. NC, normal control; DIO, diet-induced obesity; CD, control diet; HFD, high-fat diet.

Because *Acaa2*, *Acsl1* and *Cox7a1* are related to energy metabolism, we chose these three differentially methylated genes, which were found in methylation array, to perform MeDIP qPCR analysis. **Figures 7A, C, E** show CpG islands in *Acaa2*, *Acsl1* and *Cox7a1*. The gene methylation levels of *Acaa2*, *Acsl1* and *Cox7a1* were higher in the NC-HFD group than in the NC-CD group (diet exposure *P* < 0.01, **Figures 7B, D, F**). Interestingly, DIO-CD offspring had higher *Acaa2*, *Acsl1* and *Cox7a1* gene methylation levels than NC-CD offspring (prenatal exposure, *P* < 0.01, **Figures 7B, D, F**).

### The Effect of Maternal HFD on Gene Expression in Male Offspring BAT

**Figure 8:**
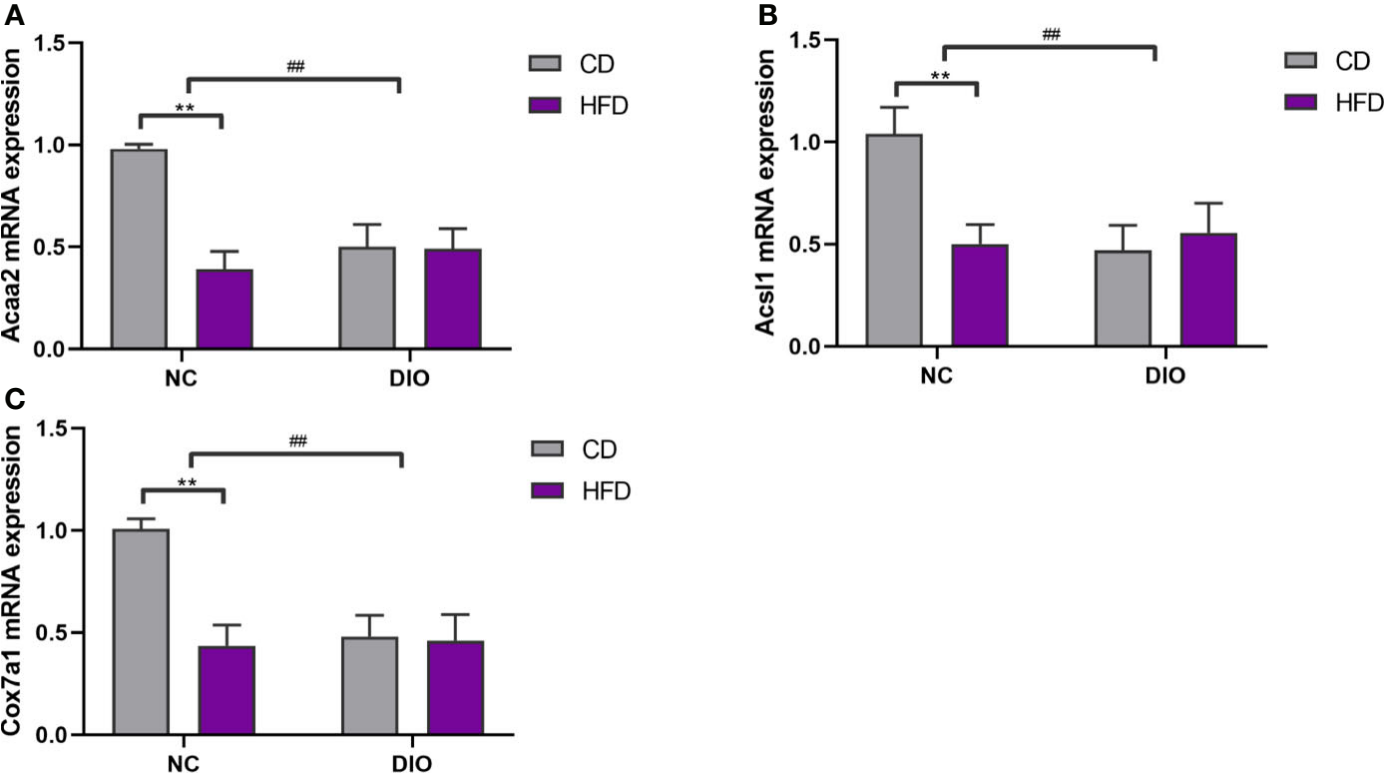
The effect of maternal HFD reduced **(A)** *Acaa2*, **(B)** *Acsl1*, **(C)** *Cox7a1* expression in male offspring BAT. Values represent the mean ± SEM, n = 8 each group. *P* values represent significance in the main effect for each source of variation (diet or prenatal exposure) as calculated by two-way ANOVA: **P < 0.01 offspring diet effect, ^##^ *P* < 0.01 maternal diet effect. NC, normal control; DIO, diet-induced obesity; CD, control diet; HFD, high-fat diet.

The expression levels of *Acaa2*, *Acsl1* and Cox7a1 were reduced in the NC-HFD offspring (diet exposure, *P* < 0.01, **Figure 8**) and offspring from DIO mothers (prenatal exposure, *P* < 0.01, **Figure 8**) compared to those in the NC-CD group.

## Discussion

In our research, although maternal HFD before and during pregnancy and lactation did not affect offspring energy intake, it increased offspring body weight at 8 and 16 weeks old. Previous studies also found that maternal HFD during this critical period elevated offspring body weight from weaning to 20 weeks old in a rodent model (38, 39). These studies highlight the long term effect of the maternal diet model on the offspring metabolic phenotype related to impaired energy metabolism. In addition, we found that pups from mothers fed a HFD had higher serum TC levels at 16 weeks old. Previous studies also found that serum TC levels were persistently higher in offspring from maternal HFD exposure than in those from maternal chow exposure at 3 weeks old (12, 40).

Moreover, our data showed that maternal HFD exposure led to an increase BAT weight in adult male offspring. In addition, the histological results of H&E staining also showed the accumulation of lipid droplets in brown adipocytes in male offspring from mothers fed a HFD. These results reveal that maternal HFD before and during pregnancy and lactation resulted in a male offspring BAT “whitening” phenotype. BAT has an important effect on energy metabolism regulation. BAT “whitening” is related to the progression of obesity. Activation of BAT is a potent target for metabolic diseases, such as diabetes and obesity (40, 41).

A previous study proved that maternal HFD induced early-onset obesity and increased lipid accumulation in BAT in weanling rats (32). The BAT of fetal mice from obese mothers had more lipid droplets, and reduced mitochondrial DNA and mitochondrial biogenesis markers (42). Offspring from mothers fed a HFD during lactation had larger brown adipocytes with the accumulation of fat droplets and increased BAT mass in adult offspring (33). Regarding the function of BAT, mitochondrial DNA mass and mitochondrial biological markers (Ndufs8, Atp5a1, and Nrf2) were reduced in the fetal BAT in mice from obese mothers (42). Moreover, maternal obesity inhibits the expression of *Drp1*, which is upstream from the transcription factor of energy metabolism regulation Forkhead box O1 (FoxO1) in fetal BAT (43). Interestingly, a normal diet after weaning only partially reverted the mitochondrial dysfunction caused by maternal HFD (43). Thus, our research provides proof that maternal HFD consumption induces BAT “whitening” in adult male offspring.

Some research has addressed the differences in the genomic methylation status between white adipose tissue and brown adipose tissue. One study observed dynamic DNA methylation changes during adipogenesis in white and brown adipose tissues. Their results revealed that there were more hypermethylated sites overall in white adipose tissue than in brown adipose tissue. Hypermethylated sites were mostly located in intronic and intergenic regions in white adipose tissue. However, hypomethylated sites in brown adipose were mostly in exonic regions. These hypomethylated promoters play a key role in the transcription of genes involved in brown fat functions, such as the mitochondrial respiratory chain and fatty acid oxidation (44). Decreased uncoupling protein-1 (UCP-1) enhancer CpG methylation is associated with high BAT-specific expression of UCP-1 (45).

Increasing evidence suggests epigenetic modification is a key mechanism in gestation and lactation programming in offspring (46, 47). Among these epigenetic mechanisms, DNA methylation has been proven in several intrauterine adverse environmental exposure models (47–50). In a rodent intrauterine growth retardation model, DNA methylation alterations were found in several key transcription factors, such as peroxisome proliferator-activated receptor-α (PPARα), glucocorticoid receptor (GR), pancreatic duodenal homeobox-1 (PDX-1), and hepatocyte nuclear factor-α (HNF-α) (46, 49, 50). In addition, maternal HFD affects the DNA methylation status of multiple tissues in offspring, including the pancreas, hypothalamus, muscle and liver (48, 51–54). Moreover, the maternal nutrition status also affects DNA methylation in adipose tissues (55, 56). Maternal obesity alters the DNA methylation of key pro-adipogenic genes, including Zfp423 and C/EBP-β, in the WAT in offspring (57). Yu et al. found increased DNA methylation of BAT-specific genes, including *Ucp1*, cytochrome c oxidase subunit (*Cox5b*), and fatty acid elongase 3 (*Elovl3*), in offspring mice from mothers with streptozotocin-induced diabetes (58). Excessive maternal glucocorticoids during pregnancy inhibited *Ppargc1a* expression through promoter DNA hypermethylation, which impaired fetal BAT function (59). In our study, we did not found *Ucp1*, *Cox5b*, *Elovl3*, and *Ppargc1a* methylation status changes in DIO-CD mice, partly because of the different maternal exposure and modeling method.

Some fatty acid oxidation (FAO)-related genes were hypermethylated in BAT from DOI-CD offspring compared to the NC-CD group. BAT mitochondria provide energy basis for thermogenesis. In mitochondria, fatty acids are a source of oxidative fuel. Unlike white adipocytes, brown adipocytes have several specific proteins that participate in FAO, such as *Acsl1* and *Acaa2*. FAO function is important for the activation and maintenance of BAT thermogenic programming (60). Enzymes involved in FAO are the switches of thermogenesis (61). After fatty acids are transported in the mitochondria, they are catalyzed to fatty acyl-CoAs by acyl-CoA synthetases, such as *Acsl1*. Then, long-chain fatty acids are transported into the mitochondrial matrix by carnitine palmitoyltransferase. In the mitochondrial matrix, acyl-CoAs are activated to acetyl-CoAs by acyl-CoA dehydrogenases. Finally, acetyl-CoA products participate in the citric acid cycle and electron transport chain. A previous study found that short term cold stimulation increased BAT-specific FAO-related gene expression in subcutaneous WAT (62). The *Acaa2* and *Acsl1* expression levels were lower in BAT of G protein-coupled receptor 120 (GPR120)-deficient mice than in BAT of normal mice (63). GPR120 is highly expressed in the BAT and cold exposure further increases its expression in BAT of mice (64). HFD-fed GPR120-deficient mice are more prone to obesity and fatty liver than wide-type mice (65). Cold exposure increases BAT-specific mitochondrial FAO enzyme expression in subcutaneous WAT, including *Acaa2* (62). *Acsl1* is also essential for the synthesis of triacylglycerol, mitochondrial function and FAO activity in brown adipocytes (66). *Acsl1*-deficient mice showed an obese phenotype and severe cold intolerance (67). We found that maternal HFD activated *Acaa2* and *Acsl1* methylation, and inhibited their expression in BAT from male offspring. This result reflects that maternal HFD-induced obesity in male offspring obesity is regarded to the inhibitory action of BAT fatty acid oxidation.

As a terminal and rate-limiting enzyme of the respiratory chain, COX7A1 is expressed in tissues that are rich in mitochondria and high in aerobic capacity in mammals, such as the heart, skeletal muscle, and BAT (68, 69). Similar to UCP1, COX also mediates BAT thermogenesis function through translocation from coupled protons to uncoupled protons in mitochondria. Cold stimulation strongly increased COX7A1 protein expression in rodent BAT (70). *Cox7a1* is a thermogenic gene, especially in a cold-responsive pattern. During cold-exposure, C57BL/6J wild-type mice showed increased COX activity and norepinephrine-induced heat production, but were unaffected in *Cox7a1* knockout mice (71). *Cox7a1* expression was increased in BAT compared with WAT. It is involved in activating brown adipocytes (72). Depletion of BAT in a rodent model increased the likelihood of HFD-induced obesity (73). Our results showed that male offspring mice from HFD dams have higher *Cox7a1* gene methylation levels, and lower *Cox7a1* expression. These results indicate that maternal HFD impairs the BAT thermogenic program in offspring.

## Conclusion

**Figure 9:**
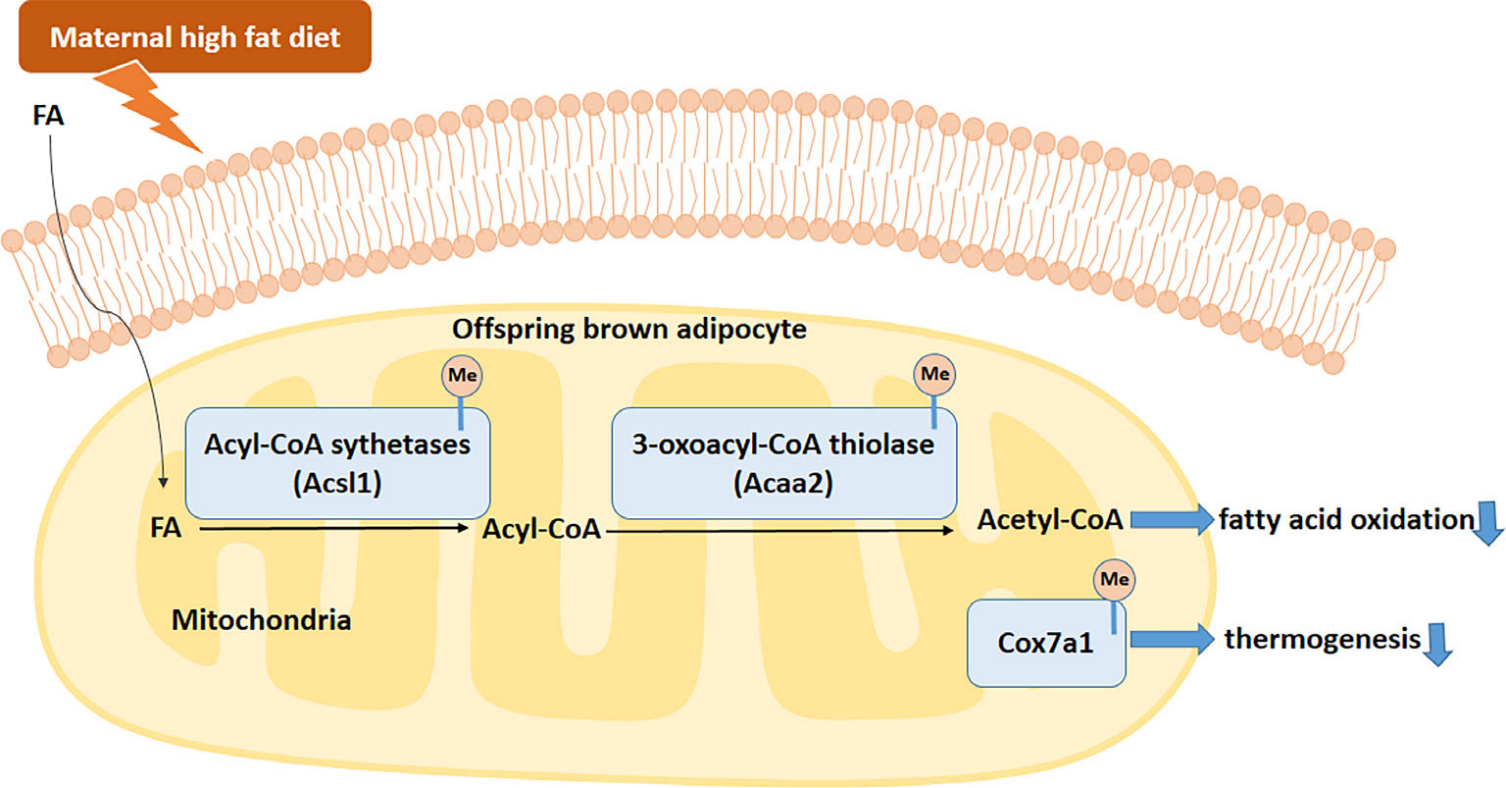
Proposed mechanism by which maternal HFD disrupts brown adipose tissue (BAT) fatty acid oxidation (FAO) and thermogenesis in male offspring. Fatty acids (FAs) enter brown adipocytes and are activated by acyl-CoA sythetases, including *Acsl1*, to form acyl-CoAs. Then acyl-CoAs were catabolized to acetyl-CoAs by acyl-CoA dehydrogenases, such as *Acaa2*. Maternal HFD active the methylation of offspring BAT FAO related enzymes, such as *Acsl1* and *Acaa2*. Meanwhile, *Cox7a1* gene methylation in offspring BAT was also active by maternal HFD. To sum up, maternal HFD disturbs the BAT FAO and thermogenesis through activated specific gene DNA methylation. FA, fatty acid.

In summary, our data suggested that maternal HFD consumption before and during pregnancy and lactation disrupts BAT structure and function. This is related to the activated methylation of *Acaa2*, *Acsl1*, and *Cox7a1* promoters. The hypermethylation of these key BAT-specific genes disturbs BAT FAO and thermogenesis and increases the risks of obesity and metabolic dysfunctions in male offspring (**Figure 9**). All of these findings uncover the transgenerational effects of maternal HFD in BAT. Specifically targeting BAT intervention during the early stage of life may become a promising strategy for metabolic diseases.

## Data Availability Statement

The datasets presented in this study can be found in online repositories. The names of the repository/repositories and accession number(s) can be found in the article/**Supplementary Material**.

## Ethics Statement

All experimental protocols were approved by the Animal Care Committee of Peking Union Medical Hospital (Permit Number: XHDW-2015-0051).

## Author Contributions

XX designed the experiments, contributed reagents and materials. QZ, JZ, TW, and XW conducted the experiments. MY, ML, and FP analyzed the data. QZ wrote the manuscript. All authors contributed to the article and approved the submitted version.

## Funding

This work was supported by the grants from National Natural Science Foundation of China (No. 81870579, 81870545, 81570715, 81170736), Beijing Natural Science Foundation (7202163), Beijing Natural Science Foundation (7202163), Beijing Municipal Science & Technology Commission (Z201100005520011), National Key R&D Program of China (2017YFC1309603), National Key Research and Development Program of China (2016YFA0101002, 2018YFC2001100), Scientific Activities Foundation for Selected Returned Overseas Professionals of Human Resources and Social Security Ministry, Beijing Dongcheng District Outstanding Talent Funding Project (2019DCT-M-05), Medical Epigenetics Research Center, Chinese Academy of Medical Sciences (2017PT31036, 2018PT31021), the Non-profit Central Research Institute Fund of Chinese Academy of Medical Sciences (No. 2017PT32020, No. 2018PT32001), Chinese Academy of Medical Sciences Innovation Fund for Medical Sciences (CIFMS2017-I2M-1-008).

## Conflict of Interest

The authors declare that the research was conducted in the absence of any commercial or financial relationships that could be construed as a potential conflict of interest.

## Publisher’s Note

All claims expressed in this article are solely those of the authors and do not necessarily represent those of their affiliated organizations, or those of the publisher, the editors and the reviewers. Any product that may be evaluated in this article, or claim that may be made by its manufacturer, is not guaranteed or endorsed by the publisher.
