# Supplementary material for: Maternal High-Fat Diet Disturbs the DNA Methylation Profile in the Brown Adipose Tissue of Offspring Mice
Source: Front Endocrinol (Lausanne). 2021 Oct 8;12:705827. doi: 10.3389/fendo.2021.705827 (PMC8531551; doi:10.3389/fendo.2021.705827)
Supplement: Supplementary file 1 [file Table_1.docx]

**Supplementary Table 1** Differentially methylated peaks (DEP) in brown adipose tissue of DIO-CD group compared with NC-CD group.

| **Peak Region** | **Gene Name** | **Peak Score** | **Peak M-value** | **Peak To TSS** |
| --- | --- | --- | --- | --- |
| 554 hypermethylated peaks | | | | |
| chr1:13650364-13650602 | Lactb2 | 2.29 | 0.065 | 107 |
| chr1:23108596-23109783 | 4933415F23Rik | 2.48 | 0.105 | -97 |
| chr1:43009039-43010064 | Gpr45 | 2.63 | 0.093 | -164 |
| chr1:74817181-74817830 | Wnt6 | 3.13 | 0.121 | -959 |
| chr1:77511215-77511693 | Epha4 | 2.48 | 0.193 | 209 |
| chr1:81072735-81073204 | 9430031J16Rik | 2.38 | 0.081 | -921 |
| chr1:88566649-88566898 | Nppc | 2.76 | 0.171 | 373 |
| chr1:127573303-127573651 | Gpr39 | 2.41 | 0.068 | -95 |
| chr1:136186049-136186762 | Myog | 2.29 | 0.166 | -174 |
| chr1:148344817-148345259 | Fam5c | 2.16 | 0.116 | 222 |
| chr1:161974115-161974570 | 4930523C07Rik | 2.59 | 0.075 | -167 |
| chr1:163180813-163181062 | Prdx6 | 2.34 | 0.129 | 359 |
| chr1:166178913-166179228 | Slc19a2 | 2.36 | 0.103 | -114 |
| chr1:172519820-172520116 | Nos1ap | 2.49 | 0.081 | 12 |
| chr1:174489726-174489981 | Vsig8 | 2.16 | 0.235 | -182 |
| chr1:174627137-174627405 | Crp | 2.3 | 0.117 | -915 |
| chr1:180267873-180268122 | Hnrnpu | 2.04 | 0.077 | -82 |
| chr1:192849872-192850109 | Mfsd7b | 2.39 | 0.091 | 78 |
| chr2:19580472-19580723 | Gm3230 | 2.04 | 0.140 | -1073 |
| chr2:25677586-25678252 | Lcn9 | 2.51 | 0.113 | -753 |
| chr2:26092445-26092699 | Qsox2 | 2.25 | 0.084 | 368 |
| chr2:26244311-26245649 | Pmpca | 2.44 | 0.077 | 113 |
| chr2:26244311-26245649 | Sdccag3 | 2.44 | 0.077 | -144 |
| chr2:29658140-29658586 | Slc27a4 | 2.56 | 0.086 | 164 |
| chr2:35056579-35056843 | Rab14 | 2.11 | 0.041 | -71 |
| chr2:35317853-35318167 | Ggta1 | 2.32 | 0.135 | -1065 |
| chr2:54288790-54289112 | Galnt13 | 2.36 | 0.134 | 154 |
| chr2:58012247-58012488 | Cytip | 2.46 | 0.075 | 165 |
| chr2:61549346-61550066 | Psmd14 | 2.51 | 0.107 | -44 |
| chr2:65076609-65077321 | Cobll1 | 2.27 | 0.051 | -282 |
| chr2:69507815-69508534 | Kbtbd10 | 3.17 | 0.137 | -1 |
| chr2:69560924-69561172 | Ppig | 2.33 | 0.110 | -96 |
| chr2:71624962-71625414 | Itga6 | 3.11 | 0.226 | 49 |
| chr2:84498982-84499523 | Gm13718 | 3.02 | 0.210 | 17 |
| chr2:84726594-84727573 | Rtn4rl2 | 2.37 | 0.093 | -234 |
| chr2:91096997-91097869 | Pacsin3 | 3.47 | 0.109 | -52 |
| chr2:91096997-91097869 | Pacsin3 | 3.47 | 0.109 | 464 |
| chr2:93174149-93174819 | Tspan18 | 3.15 | 0.144 | 160 |
| chr2:119373545-119374414 | 1500003O03Rik | 2.58 | 0.145 | 537 |
| chr2:119373545-119374414 | Exd1 | 2.58 | 0.145 | -616 |
| chr2:127617725-127618441 | 1500011K16Rik | 2.07 | 0.108 | 141 |
| chr2:143688423-143689291 | Bfsp1 | 2.92 | 0.156 | 52 |
| chr2:157173223-157173685 | Ghrh | 2.63 | 0.118 | -1063 |
| chr2:167457523-167458005 | Ube2v1 | 2.42 | 0.155 | -259 |
| chr2:168032558-168032856 | Adnp | 2.97 | 0.110 | -145 |
| chr2:173346844-173347312 | 1700021F07Rik | 2.72 | 0.210 | -1014 |
| chr2:180376912-180377673 | Tcfl5 | 2.78 | 0.104 | 103 |
| chr3:34547630-34548977 | Sox2 | 2.79 | 0.098 | -622 |
| chr3:36049955-36050409 | D3Ertd254e | 2.34 | 0.161 | 181 |
| chr3:51219850-51220127 | Naa15 | 2.32 | 0.080 | 51 |
| chr3:54538996-54539526 | Alg5 | 2.98 | 0.148 | -199 |
| chr3:54538996-54539526 | Exosc8 | 2.98 | 0.148 | 25 |
| chr3:54858210-54859685 | Ccna1 | 2.62 | 0.073 | 29 |
| chr3:63768157-63768625 | Slc33a1 | 2.37 | 0.094 | 264 |
| chr3:79431701-79432174 | 4930579G24Rik | 2.43 | 0.098 | -1062 |
| chr3:84386058-84387337 | Arfip1 | 2.77 | 0.111 | -150 |
| chr3:89017759-89018730 | Mtx1 | 2.91 | 0.107 | 12 |
| chr3:89017759-89018730 | Thbs3 | 2.91 | 0.107 | -863 |
| chr3:90017074-90017729 | Gm9846 | 2.54 | 0.083 | 167 |
| chr3:90017074-90017729 | Rps27 | 2.54 | 0.083 | 168 |
| chr3:95462077-95462661 | Mcl1 | 2.76 | 0.107 | -273 |
| chr3:96072270-96072931 | Hist2h2bb | 2.76 | 0.312 | -1021 |
| chr3:96072270-96072931 | Hist2h3b | 2.76 | 0.312 | -15 |
| chr3:102538591-102539134 | Tspan2 | 2.75 | 0.150 | 170 |
| chr3:107434337-107435505 | Fam40a | 2.4 | 0.076 | -293 |
| chr3:125641394-125641937 | Ugt8a | 2.53 | 0.071 | -197 |
| chr3:129672640-129673094 | Ccdc109b | 2.75 | 0.155 | 257 |
| chr3:136332653-136332903 | Ppp3ca | 2.18 | 0.085 | -955 |
| chr3:144234033-144234291 | Hs2st1 | 2.69 | 0.147 | -982 |
| chr3:145587365-145587610 | Bcl10 | 2.71 | 0.147 | 146 |
| chr4:19968868-19969608 | Ggh | 2.6 | 0.135 | 40 |
| chr4:41702060-41702298 | Galt | 2.29 | 0.093 | 54 |
| chr4:48675968-48676764 | Murc | 2.63 | 0.097 | -19 |
| chr4:52836983-52838117 | Olfr275 | 3.01 | 0.137 | -720 |
| chr4:57155544-57157121 | Epb4.1l4b | 3 | 0.165 | -304 |
| chr4:63064951-63065683 | Akna | 2.93 | 0.132 | -838 |
| chr4:71860987-71861432 | C630043F03Rik | 2.38 | 0.127 | -1067 |
| chr4:94269727-94270417 | Plaa | 2.29 | 0.063 | -134 |
| chr4:96452696-96452944 | Gm12695 | 2.3 | 0.084 | -969 |
| chr4:101389702-101390160 | Lepr | 2.82 | 0.228 | -80 |
| chr4:106880895-106881621 | Ldlrad1 | 2.17 | 0.134 | -526 |
| chr4:117222970-117223216 | Eri3 | 2.17 | 0.078 | -5 |
| chr4:125168150-125168394 | Grik3 | 2.28 | 0.190 | 198 |
| chr4:125999143-125999657 | Adprhl2 | 2.14 | 0.111 | -453 |
| chr4:126429706-126430077 | AU040320 | 2.2 | 0.094 | -906 |
| chr4:126429706-126430077 | AU040320 | 2.2 | 0.094 | -1121 |
| chr4:127648307-127648759 | CK137956 | 2.16 | 0.101 | -448 |
| chr4:129349729-129350171 | Kpna6 | 2.74 | 0.142 | 61 |
| chr4:130125058-130125305 | Nkain1 | 2.23 | 0.121 | -522 |
| chr4:130218895-130219345 | Pum1 | 2.34 | 0.063 | -153 |
| chr4:132288780-132289440 | Xkr8 | 2.47 | 0.139 | -649 |
| chr4:132686377-132686843 | Wasf2 | 2.6 | 0.085 | 63 |
| chr4:133310255-133310774 | Arid1a | 2.52 | 0.141 | -988 |
| chr4:133524433-133524743 | Hmgn2 | 2.38 | 0.093 | -682 |
| chr4:135182534-135182987 | 1700029M20Rik | 2.45 | 0.140 | 191 |
| chr4:135282789-135283536 | Il22ra1 | 3.01 | 0.213 | -971 |
| chr4:135728379-135728624 | E2f2 | 2.18 | 0.077 | 193 |
| chr4:136604130-136604798 | Zbtb40 | 2.58 | 0.074 | 146 |
| chr4:140565905-140566640 | Mfap2 | 2.22 | 0.085 | -65 |
| chr4:140565905-140566640 | Mfap2 | 2.22 | 0.085 | -278 |
| chr4:140703487-140704178 | Fbxo42 | 2.26 | 0.120 | -3 |
| chr4:141219773-141220480 | Plekhm2 | 2.35 | 0.133 | -96 |
| chr4:141430337-141430587 | Efhd2 | 2.49 | 0.149 | 373 |
| chr4:147242703-147242947 | Fv1 | 2.68 | 0.060 | -262 |
| chr4:147242703-147242947 | Miip | 2.68 | 0.060 | 3 |
| chr4:149037122-149038225 | Pik3cd | 2.51 | 0.134 | -248 |
| chr4:149493353-149493868 | Slc2a5 | 2.54 | 0.132 | 158 |
| chr4:151665508-151666887 | Hes3 | 2.83 | 0.106 | -426 |
| chr4:153400155-153400826 | 1190007F08Rik | 2.24 | 0.110 | -337 |
| chr4:154230041-154230311 | Ttc34 | 2.08 | 0.060 | -132 |
| chr4:154334764-154335416 | Hes5 | 2.46 | 0.101 | 59 |
| chr4:154336950-154337193 | Pank4 | 2.6 | 0.220 | -1169 |
| chr4:155042868-155043343 | Mib2 | 2.39 | 0.165 | 230 |
| chr4:155213131-155213787 | Mxra8 | 2.34 | 0.207 | -329 |
| chr5:7344929-7345380 | Zfp804b | 2.49 | 0.202 | -776 |
| chr5:20387796-20388045 | Phtf2 | 2.15 | 0.059 | 21 |
| chr5:20387796-20388045 | Tmem60 | 2.15 | 0.059 | -349 |
| chr5:23929113-23929785 | Cdk5 | 2.69 | 0.071 | -101 |
| chr5:23929113-23929785 | Slc4a2 | 2.69 | 0.071 | -1600 |
| chr5:31597960-31598427 | Fndc4 | 2.39 | 0.070 | 56 |
| chr5:33617405-33618138 | Ctbp1 | 2.8 | 0.081 | -118 |
| chr5:34885329-34885792 | Sh3bp2 | 2.33 | 0.054 | -452 |
| chr5:35003052-35003931 | Nop14 | 3.19 | 0.169 | -694 |
| chr5:35003052-35003931 | Grk4 | 3.19 | 0.169 | 464 |
| chr5:35868596-35869058 | Cpz | 2.43 | 0.063 | -552 |
| chr5:37221400-37222061 | Man2b2 | 2.86 | 0.156 | 157 |
| chr5:48373355-48374888 | Slit2 | 3.05 | 0.131 | -271 |
| chr5:68238801-68239460 | Atp8a1 | 2.32 | 0.292 | -460 |
| chr5:73683771-73684496 | Ociad1 | 2.6 | 0.134 | 101 |
| chr5:73871458-73872661 | Dcun1d4 | 2.79 | 0.093 | -233 |
| chr5:74487825-74489364 | Snora26 | 2.73 | 0.084 | -959 |
| chr5:74487825-74489364 | 2700023E23Rik | 2.73 | 0.084 | -512 |
| chr5:77524823-77525291 | Hopx | 2.61 | 0.207 | -765 |
| chr5:106305439-106305899 | Zfp326 | 2.42 | 0.116 | 83 |
| chr5:108153410-108154684 | Gfi1 | 2.46 | 0.077 | -684 |
| chr5:108416222-108416475 | Fam69a | 2.47 | 0.062 | -252 |
| chr5:108978547-108979216 | Cplx1 | 2.59 | 0.104 | 164 |
| chr5:109123043-109123517 | Fgfrl1 | 2.13 | 0.136 | 33 |
| chr5:109987525-109987969 | Crlf2 | 2.25 | 0.134 | 265 |
| chr5:111081379-111082996 | Ddx51 | 2.55 | 0.083 | -281 |
| chr5:111081379-111082996 | Noc4l | 2.55 | 0.083 | 213 |
| chr5:117565551-117566496 | Suds3 | 2.57 | 0.186 | -21 |
| chr5:123156286-123156535 | P2rx4 | 2.74 | 0.143 | -1154 |
| chr5:124980903-124981712 | Rilpl1 | 2.47 | 0.096 | 92 |
| chr5:129105694-129106735 | 5930412G12Rik | 3.21 | 0.103 | 347 |
| chr5:129105694-129106735 | Fzd10 | 3.21 | 0.103 | -765 |
| chr5:130845006-130845661 | Caln1 | 2.9 | 0.193 | 6 |
| chr5:139230499-139231499 | Fam20c | 2.34 | 0.126 | -35 |
| chr5:139801555-139802634 | Adap1 | 2.68 | 0.172 | -676 |
| chr5:140980687-140981273 | Chst12 | 2.08 | 0.093 | -582 |
| chr5:143721863-143722102 | Fscn1 | 2.54 | 0.047 | -50 |
| chr5:144090159-144090680 | 0610040B10Rik | 2.24 | 0.064 | -566 |
| chr5:144090159-144090680 | Zdhhc4 | 2.24 | 0.064 | 485 |
| chr5:147887827-147888293 | Lnx2 | 2.5 | 0.105 | 88 |
| chr5:147887827-147888293 | Polr1d | 2.5 | 0.105 | -1056 |
| chr5:147887827-147888293 | Polr1d | 2.5 | 0.105 | -1083 |
| chr5:148768353-148769070 | Mtus2 | 2.67 | 0.079 | -183 |
| chr6:30688040-30688485 | Mest | 2.83 | 0.273 | 200 |
| chr6:34267160-34267618 | Akr1b3 | 2.47 | 0.086 | 100 |
| chr6:35083253-35083707 | Cnot4 | 2.72 | 0.222 | 257 |
| chr6:38383684-38384125 | Ubn2 | 2.59 | 0.199 | -19 |
| chr6:39067812-39068064 | Parp12 | 2.46 | 0.094 | 410 |
| chr6:52158343-52158786 | Hoxa6 | 2.41 | 0.174 | 58 |
| chr6:54277107-54277347 | Prr15 | 2.41 | 0.095 | 222 |
| chr6:57641583-57641834 | Pigy | 2.5 | 0.131 | 363 |
| chr6:83660853-83661348 | Vax2os1 | 2.37 | 0.236 | -174 |
| chr6:83660853-83661348 | Vax2 | 2.37 | 0.236 | -156 |
| chr6:83969027-83969678 | Dysf | 2.34 | 0.135 | -29 |
| chr6:84544817-84545080 | Cyp26b1 | 2.57 | 0.158 | -1144 |
| chr6:84544817-84545080 | Cyp26b1 | 2.57 | 0.158 | -1046 |
| chr6:86320917-86321354 | Snrpg | 2.11 | 0.154 | -397 |
| chr6:88674701-88674947 | Mgll | 2.51 | 0.148 | 419 |
| chr6:88674701-88674947 | Mgll | 2.51 | 0.148 | 127 |
| chr6:90217812-90218102 | Vmn1r54 | 2.69 | 0.131 | -1142 |
| chr6:108609839-108610094 | Bhlhe40 | 2.59 | 0.112 | -655 |
| chr6:112337928-112338190 | D630042P16Rik | 2.05 | 0.041 | -42 |
| chr6:122258691-122259357 | M6pr | 2.44 | 0.145 | -3 |
| chr6:122656769-122657705 | Nanogpd | 2.84 | 0.076 | -563 |
| chr6:122656769-122657705 | Nanog | 2.84 | 0.076 | -348 |
| chr6:124365010-124365726 | Pex5 | 2.07 | 0.075 | -283 |
| chr6:124778902-124779970 | Cdca3 | 3.07 | 0.164 | -757 |
| chr6:124778902-124779970 | Usp5 | 3.07 | 0.164 | 29 |
| chr6:126871465-126872564 | Dyrk4 | 2.52 | 0.163 | -157 |
| chr6:135117767-135118453 | Hebp1 | 2.46 | 0.173 | 123 |
| chr6:135259913-135260603 | Pbp2 | 2.42 | 0.112 | 144 |
| chr6:143115073-143115315 | Etnk1 | 2.19 | 0.160 | -555 |
| chr6:147212320-147212563 | Pthlh | 2.17 | 0.067 | 165 |
| chr6:148303331-148303565 | Rps4y2 | 2.27 | 0.157 | 271 |
| chr7:5077478-5077724 | Rasl2-9-ps | 2.61 | 0.076 | -49 |
| chr7:16683851-16684097 | Napa | 2.58 | 0.072 | -17 |
| chr7:19609211-19610165 | Foxa3 | 2.74 | 0.124 | -800 |
| chr7:19609211-19610165 | Sympk | 2.74 | 0.124 | -37 |
| chr7:19661461-19661946 | Dmwd | 2.59 | 0.253 | 155 |
| chr7:26090328-26090577 | Tmem145 | 2.7 | 0.085 | -673 |
| chr7:30968816-30969078 | Cox7a1 | 2.05 | 0.084 | -242 |
| chr7:31646207-31646456 | Ffar1 | 2.21 | 0.094 | 157 |
| chr7:35971025-35971831 | Slc7a10 | 3.54 | 0.166 | 25 |
| chr7:38968257-38968922 | 1600014C10Rik | 2.48 | 0.162 | 354 |
| chr7:38968257-38968922 | 1600014C10Rik | 2.48 | 0.162 | -215 |
| chr7:52459975-52460227 | Ccdc155 | 2.82 | 0.138 | 161 |
| chr7:52625447-52626308 | Lin7b | 2.76 | 0.105 | 56 |
| chr7:56044169-56044824 | Zdhhc13 | 3.28 | 0.151 | 124 |
| chr7:85722430-85723592 | Ntrk3 | 2.51 | 0.127 | -287 |
| chr7:87439083-87439746 | Prc1 | 2.44 | 0.094 | 64 |
| chr7:87487668-87487959 | Hddc3 | 2.51 | 0.111 | -208 |
| chr7:88908048-88908311 | Fam103a1 | 2.51 | 0.201 | 341 |
| chr7:89136686-89136936 | Bnc1 | 2.09 | 0.147 | 374 |
| chr7:105804595-105805252 | 2210018M11Rik | 2.44 | 0.156 | 155 |
| chr7:109358292-109358959 | Nup98 | 2.5 | 0.083 | 8 |
| chr7:109398971-109399433 | Rhog | 2.31 | 0.053 | -570 |
| chr7:109589897-109590553 | Rrm1 | 2.89 | 0.143 | 17 |
| chr7:138009212-138009458 | Plekha1 | 2.34 | 0.050 | -88 |
| chr7:150481965-150482827 | Kcnq1ot1 | 3.95 | 0.211 | 56 |
| chr8:10927958-10928906 | 3930402G23Rik | 3.17 | 0.109 | 25 |
| chr8:12914593-12915969 | Mcf2l | 2.89 | 0.123 | -611 |
| chr8:13025563-13026061 | F7 | 2.53 | 0.051 | -221 |
| chr8:13288375-13288831 | Dcun1d2 | 2.65 | 0.049 | -477 |
| chr8:13288375-13288831 | Tmco3 | 2.65 | 0.049 | 591 |
| chr8:13678124-13678571 | Rasa3 | 2.53 | 0.101 | -760 |
| chr8:41596785-41597299 | Cnot7 | 2.77 | 0.111 | -384 |
| chr8:41596785-41597299 | Vps37a | 2.77 | 0.111 | -94 |
| chr8:47555098-47556716 | Acsl1 | 3.6 | 0.123 | -488 |
| chr8:48760942-48761605 | Ing2 | 2.44 | 0.101 | -760 |
| chr8:59966568-59967102 | Sap30 | 2.41 | 0.063 | -178 |
| chr8:63368920-63369168 | 2700029M09Rik | 2.25 | 0.066 | -203 |
| chr8:72177163-72177643 | Gm7187 | 3.26 | 0.188 | -1051 |
| chr8:73246729-73248429 | Pde4c | 3.96 | 0.160 | -383 |
| chr8:74195190-74196049 | Unc13a | 3.45 | 0.150 | 36 |
| chr8:75877294-75877748 | Large | 2.85 | 0.114 | -1066 |
| chr8:87605206-87605875 | Man2b1 | 2.1 | 0.088 | -1626 |
| chr8:87605206-87605875 | BC056474 | 2.1 | 0.088 | 679 |
| chr8:87605206-87605875 | Wdr83 | 2.1 | 0.088 | -895 |
| chr8:88079081-88079625 | Dnaja2 | 2.64 | 0.125 | -183 |
| chr8:96334668-96334951 | Gnao1 | 2.18 | 0.165 | 72 |
| chr8:96852979-96853433 | Slc12a3 | 2.71 | 0.138 | 99 |
| chr8:107788734-107788974 | Fbxl8 | 2.3 | 0.093 | 307 |
| chr8:107788734-107788974 | Tradd | 2.3 | 0.093 | -360 |
| chr8:107792621-107792861 | Hsf4 | 2.52 | 0.080 | -1032 |
| chr8:107898486-107899359 | Plekhg4 | 2.44 | 0.067 | -357 |
| chr8:108375423-108376378 | Cenpt | 3.17 | 0.125 | 7 |
| chr8:109127251-109127497 | Cdh1 | 2.16 | 0.063 | 107 |
| chr8:109579825-109580477 | Cog8 | 2.59 | 0.165 | 486 |
| chr8:109579825-109580477 | Nip7 | 2.59 | 0.165 | -625 |
| chr8:113823636-113824350 | Rfwd3 | 2.56 | 0.110 | 129 |
| chr8:114464582-114464865 | Gabarapl2 | 2.58 | 0.086 | 106 |
| chr8:131588901-131589142 | Ccdc7 | 2.65 | 0.153 | 370 |
| chr9:14574977-14575695 | 1700012B09Rik | 2.77 | 0.147 | 138 |
| chr9:25288497-25288756 | Eepd1 | 2.15 | 0.145 | -554 |
| chr9:43909305-43910169 | Mfrp | 2.27 | 0.209 | -115 |
| chr9:43909305-43910169 | C1qtnf5 | 2.27 | 0.209 | -115 |
| chr9:43921045-43921923 | Rnf26 | 2.63 | 0.167 | -350 |
| chr9:44142540-44143210 | H2afx | 2.79 | 0.123 | 78 |
| chr9:44412575-44413002 | Ddx6 | 2.24 | 0.092 | -185 |
| chr9:51573170-51573574 | Arhgap20 | 2.05 | 0.114 | -84 |
| chr9:51771564-51771801 | Fdx1 | 2.2 | 0.047 | -44 |
| chr9:54546766-54547420 | Dnaja4 | 2.24 | 0.105 | -272 |
| chr9:59333682-59334427 | Arih1 | 2.84 | 0.131 | 126 |
| chr9:77391993-77393446 | Lrrc1 | 2.09 | 0.098 | -853 |
| chr9:77391993-77393446 | Lrrc1 | 2.09 | 0.098 | -60 |
| chr9:78462996-78463668 | Cd109 | 2.97 | 0.178 | -20 |
| chr9:88221523-88222775 | Nt5e | 3.32 | 0.115 | -297 |
| chr9:91263410-91263682 | Zic4 | 2.49 | 0.076 | -263 |
| chr9:97933098-97934086 | Clstn2 | 3.02 | 0.109 | -6 |
| chr9:100936124-100936593 | Pccb | 2.48 | 0.134 | -1064 |
| chr9:105711487-105712998 | Col6a6 | 3.18 | 0.162 | -264 |
| chr9:108962097-108963374 | Trex1 | 3.14 | 0.119 | -498 |
| chr9:108962097-108963374 | Trex1 | 3.14 | 0.119 | -970 |
| chr9:110527024-110528096 | Nradd | 3.69 | 0.170 | -663 |
| chr9:110644621-110644878 | Pth1r | 2.39 | 0.176 | 364 |
| chr9:119011621-119012060 | Dlec1 | 2.44 | 0.102 | 245 |
| chr9:119487956-119489110 | Scn5a | 3.07 | 0.104 | -399 |
| chr9:119932263-119933276 | Xirp1 | 3.03 | 0.146 | -53 |
| chr9:121701071-121701322 | Hhatl | 2.89 | 0.051 | 428 |
| chr9:121701071-121701322 | Hhatl | 2.89 | 0.051 | -958 |
| chr9:121824929-121825598 | Cyp8b1 | 2.6 | 0.164 | 159 |
| chr9:123387920-123388171 | Limd1 | 2.45 | 0.118 | 227 |
| chr10:12581957-12582618 | Utrn | 2.9 | 0.106 | -754 |
| chr10:23669448-23669701 | Taar3 | 2.43 | 0.156 | 211 |
| chr10:40790894-40791140 | Gpr6 | 2.18 | 0.090 | 373 |
| chr10:43988203-43988518 | Atg5 | 2.28 | 0.095 | 197 |
| chr10:58275091-58275746 | Sh3rf3 | 2.62 | 0.096 | -687 |
| chr10:61911963-61912427 | Supv3l1 | 2.47 | 0.100 | 246 |
| chr10:61911963-61912427 | 4930507D05Rik | 2.47 | 0.100 | 288 |
| chr10:62113858-62114527 | Ddx50 | 2.32 | 0.046 | -246 |
| chr10:62563063-62563317 | Atoh7 | 2.36 | 0.090 | 287 |
| chr10:70621898-70622696 | Bicc1 | 3.32 | 0.201 | 85 |
| chr10:75236440-75237371 | Ddt | 2.38 | 0.125 | -786 |
| chr10:75284789-75285668 | Gstt4 | 2.67 | 0.112 | 59 |
| chr10:75384281-75384519 | Smarcb1 | 2.08 | 0.077 | -41 |
| chr10:77068573-77068835 | Sumo3 | 2.06 | 0.068 | -274 |
| chr10:77084499-77085360 | Ube2g2 | 2.24 | 0.080 | -135 |
| chr10:79578596-79578845 | Stk11 | 2.2 | 0.143 | -559 |
| chr10:80204674-80205336 | Ap3d1 | 2.98 | 0.182 | -49 |
| chr10:80380503-80381053 | Lmnb2 | 2.45 | 0.115 | 212 |
| chr10:86955967-86956636 | Ascl1 | 3.21 | 0.176 | 103 |
| chr10:94407709-94408377 | Plxnc1 | 3.14 | 0.183 | -831 |
| chr10:94977897-94978208 | Ube2n | 2.35 | 0.234 | 257 |
| chr10:99221793-99222068 | Csl | 2.34 | 0.278 | 361 |
| chr10:116333438-116333685 | Gm239 | 2.67 | 0.146 | 373 |
| chr10:120335530-120336872 | Msrb3 | 2.8 | 0.124 | -174 |
| chr10:122116042-122116291 | Ppm1h | 2.29 | 0.122 | 349 |
| chr10:127103543-127103999 | Nab2 | 2.06 | 0.061 | -12 |
| chr10:128027015-128027456 | Erbb3 | 2.54 | 0.156 | -678 |
| chr11:3352054-3352313 | Rnf185 | 2.07 | 0.148 | 144 |
| chr11:3352054-3352313 | 8430429K09Rik | 2.07 | 0.148 | -255 |
| chr11:4964954-4965607 | Gas2l1 | 2.51 | 0.087 | 49 |
| chr11:5761642-5762355 | Aebp1 | 2.68 | 0.143 | 130 |
| chr11:6526068-6526317 | Tbrg4 | 2.28 | 0.061 | -122 |
| chr11:22760591-22760901 | B3gnt2 | 2.38 | 0.139 | -410 |
| chr11:22760591-22760901 | B3gnt2 | 2.38 | 0.139 | -1011 |
| chr11:43341578-43342516 | Ccnjl | 2.37 | 0.091 | -238 |
| chr11:48961723-48962198 | Olfr1395 | 2.53 | 0.200 | 200 |
| chr11:49944105-49944351 | Tbc1d9b | 2.26 | 0.082 | -633 |
| chr11:58790800-58791872 | Trim11 | 2.75 | 0.111 | -281 |
| chr11:59623317-59623856 | Flcn | 2.46 | 0.104 | -311 |
| chr11:61496824-61498191 | Fam83g | 2.78 | 0.065 | -403 |
| chr11:62094719-62095306 | Ttc19 | 2.62 | 0.173 | 38 |
| chr11:62094719-62095306 | Zswim7 | 2.62 | 0.173 | -115 |
| chr11:69749111-69749863 | Ybx2 | 2.49 | 0.110 | 87 |
| chr11:72174969-72176073 | Tekt1 | 3.35 | 0.155 | -139 |
| chr11:72420214-72420664 | Ube2g1 | 2.18 | 0.111 | -323 |
| chr11:75609285-75609535 | Doc2b | 2.44 | 0.138 | 149 |
| chr11:80114442-80114684 | Rhbdl3 | 2.49 | 0.194 | 150 |
| chr11:81386555-81387003 | 1700071K01Rik | 3.15 | 0.262 | 262 |
| chr11:84683825-84684400 | Ggnbp2 | 3.01 | 0.151 | 127 |
| chr11:88579264-88579777 | Msi2 | 2.54 | 0.090 | 22 |
| chr11:88725454-88726223 | Akap1 | 2.78 | 0.144 | 61 |
| chr11:88835361-88835639 | Coil | 2.93 | 0.182 | 252 |
| chr11:90499808-90500043 | Stxbp4 | 2.34 | 0.066 | -503 |
| chr11:90499808-90500043 | Cox11 | 2.34 | 0.066 | 428 |
| chr11:94071774-94072248 | Tob1 | 2.29 | 0.084 | -756 |
| chr11:94104329-94104591 | Wfikkn2 | 2.54 | 0.093 | -567 |
| chr11:96326542-96326781 | Gm11529 | 2.49 | 0.094 | -800 |
| chr11:97560457-97561401 | Pcgf2 | 3.53 | 0.182 | 882 |
| chr11:97560457-97561401 | Pcgf2 | 3.53 | 0.182 | 33 |
| chr11:97560457-97561401 | Pcgf2 | 3.53 | 0.182 | -231 |
| chr11:98307063-98308528 | Grb7 | 2.44 | 0.079 | -351 |
| chr11:98798761-98799033 | Rara | 2.02 | 0.204 | -112 |
| chr11:98798761-98799033 | Rara | 2.02 | 0.204 | -134 |
| chr11:99235718-99237047 | Krt28 | 3.55 | 0.144 | -165 |
| chr11:100389055-100389314 | Acly | 2.54 | 0.084 | 30 |
| chr11:100831433-100832345 | Ptrf | 2.62 | 0.133 | 42 |
| chr11:101228460-101229376 | G6pc | 3.49 | 0.184 | -125 |
| chr11:102268537-102269006 | Slc25a39 | 2.3 | 0.166 | 59 |
| chr11:102961753-102962007 | Acbd4 | 2.46 | 0.126 | -1121 |
| chr11:114529672-114530342 | Rpl38 | 2.14 | 0.173 | -87 |
| chr11:114529672-114530342 | Rpl38 | 2.14 | 0.173 | 151 |
| chr11:115674613-115674872 | Caskin2 | 2.11 | 0.135 | 163 |
| chr11:115674613-115674872 | Tsen54 | 2.11 | 0.135 | -1309 |
| chr11:117830825-117831336 | Socs3 | 2.01 | 0.050 | -400 |
| chr11:119409072-119409971 | Nptx1 | 2.49 | 0.069 | -387 |
| chr11:121007223-121007897 | Tex19.1 | 3.6 | 0.200 | 104 |
| chr12:8308671-8308951 | Gdf7 | 2.16 | 0.046 | -51 |
| chr12:16901765-16902260 | Rock2 | 2.84 | 0.141 | 229 |
| chr12:17272552-17273706 | Pdia6 | 3.1 | 0.198 | -271 |
| chr12:25515984-25516868 | Mboat2 | 2.47 | 0.104 | -37 |
| chr12:32319250-32319799 | Bcap29 | 2.54 | 0.071 | -62 |
| chr12:32319250-32319799 | Bcap29 | 2.54 | 0.071 | -1 |
| chr12:52930242-52930478 | Hectd1 | 2.51 | 0.233 | 163 |
| chr12:55757549-55757998 | 1110002B05Rik | 2.26 | 0.045 | -214 |
| chr12:73336803-73337245 | Rtn1 | 2.62 | 0.165 | 687 |
| chr12:77355344-77356486 | Mthfd1 | 2.9 | 0.153 | -303 |
| chr12:80292523-80292817 | Rdh11 | 2.2 | 0.079 | 136 |
| chr12:81861210-81861889 | Gm1568 | 3.02 | 0.165 | 152 |
| chr12:86216960-86217219 | Ltbp2 | 2.72 | 0.169 | 355 |
| chr12:86216960-86217219 | D030025P21Rik | 2.72 | 0.169 | 338 |
| chr12:99976005-99976464 | Ptpn21 | 2.69 | 0.072 | -619 |
| chr12:112277599-112278037 | Rcor1 | 2.05 | 0.116 | -190 |
| chr12:117314899-117315772 | Vipr2 | 2.7 | 0.107 | -859 |
| chr12:119081663-119082560 | Cdca7l | 2.82 | 0.146 | -221 |
| chr13:14722530-14722983 | AW209491 | 2.88 | 0.118 | 245 |
| chr13:14722530-14722983 | AW209491 | 2.88 | 0.118 | 232 |
| chr13:21813378-21813625 | Hist1h2bm | 2.15 | 0.100 | -464 |
| chr13:23829662-23831255 | Hist1h1c | 3.41 | 0.145 | -216 |
| chr13:46060253-46060935 | Atxn1 | 2.65 | 0.227 | -249 |
| chr13:47216844-47217099 | Rnf144b | 2.38 | 0.097 | -1116 |
| chr13:48758436-48758695 | Barx1 | 2.36 | 0.129 | 161 |
| chr13:51940797-51941258 | Gadd45g | 2.15 | 0.092 | -1015 |
| chr13:53024971-53025221 | Auh | 2.09 | 0.162 | -50 |
| chr13:54605123-54605387 | 4732471D19Rik | 2.28 | 0.096 | 90 |
| chr13:55205641-55207047 | Zfp346 | 2.51 | 0.115 | -325 |
| chr13:55815653-55816320 | Txndc15 | 2.51 | 0.132 | -23 |
| chr13:56710168-56710621 | Tgfbi | 2.48 | 0.069 | -568 |
| chr13:58009607-58010768 | Spock1 | 3.26 | 0.167 | -494 |
| chr13:81850555-81851012 | Mblac2 | 2.65 | 0.116 | 368 |
| chr13:81850555-81851012 | Polr3g | 2.65 | 0.116 | -771 |
| chr13:85329234-85329497 | Ccnh | 2.07 | 0.072 | 284 |
| chr13:99695844-99696733 | Tnpo1 | 3.02 | 0.117 | 50 |
| chr13:101322170-101322628 | Ocln | 2.76 | 0.084 | 54 |
| chr13:101386473-101386939 | Marveld2 | 2.35 | 0.201 | 193 |
| chr13:101386473-101386939 | Marveld2 | 2.35 | 0.201 | 220 |
| chr13:120276971-120278429 | Gm7120 | 2.79 | 0.170 | 855 |
| chr13:120276971-120278429 | 3110070M22Rik | 2.79 | 0.170 | -509 |
| chr13:120276971-120278429 | Gm7120 | 2.79 | 0.170 | 34 |
| chr14:16271208-16271469 | Lrrc3b | 2.3 | 0.146 | 162 |
| chr14:22317705-22319150 | Myst4 | 2.97 | 0.111 | -647 |
| chr14:22808952-22809200 | Zfp503 | 2.11 | 0.047 | -253 |
| chr14:37781658-37781909 | Gcap14 | 2.39 | 0.066 | 166 |
| chr14:55050481-55050803 | Mmp14 | 2.28 | 0.091 | 202 |
| chr14:56128261-56128976 | Cpne6 | 2.47 | 0.080 | -665 |
| chr14:56128261-56128976 | Cpne6 | 2.47 | 0.080 | -1577 |
| chr14:65977182-65977422 | Zfp395 | 2.02 | 0.067 | -210 |
| chr14:67529157-67529620 | Pnma2 | 2.24 | 0.090 | -655 |
| chr14:67627222-67627559 | Bnip3l | 2.46 | 0.103 | 323 |
| chr14:70920516-70921195 | Bmp1 | 3.04 | 0.215 | -788 |
| chr14:80170914-80171355 | Pcdh8 | 2.48 | 0.089 | -15 |
| chr14:122932783-122933665 | Pcca | 2.93 | 0.141 | -325 |
| chr15:6823814-6824688 | Osmr | 2.98 | 0.178 | 62 |
| chr15:34012059-34012314 | Mtdh | 2.27 | 0.099 | -286 |
| chr15:38837652-38837910 | Fzd6 | 2.03 | 0.036 | -44 |
| chr15:38837652-38837910 | Fzd6 | 2.03 | 0.036 | -97 |
| chr15:38943906-38944568 | Dcaf13 | 2.76 | 0.143 | -182 |
| chr15:38943906-38944568 | Slc25a32 | 2.76 | 0.143 | 25 |
| chr15:41620762-41621036 | Oxr1 | 2.06 | 0.136 | -161 |
| chr15:41620762-41621036 | Oxr1 | 2.06 | 0.136 | 320 |
| chr15:58719944-58720676 | Rnf139 | 2.45 | 0.094 | -473 |
| chr15:58913521-58913787 | Mtss1 | 2.42 | 0.059 | -73 |
| chr15:66722950-66723210 | Wisp1 | 2.34 | 0.132 | 126 |
| chr15:72640259-72641336 | Peg13 | 3.23 | 0.163 | -43 |
| chr15:75578467-75579634 | Mafa | 2.38 | 0.119 | -698 |
| chr15:76880772-76881022 | Mb | 2.49 | 0.194 | 201 |
| chr15:77787587-77788259 | Foxred2 | 2.59 | 0.179 | -771 |
| chr15:79518223-79518471 | Josd1 | 2.26 | 0.141 | -45 |
| chr15:81415359-81416447 | Ep300 | 2.83 | 0.103 | -740 |
| chr15:81845499-81846159 | Xrcc6 | 2.56 | 0.112 | -969 |
| chr15:81845499-81846159 | Pppde2 | 2.56 | 0.112 | 741 |
| chr15:94234293-94235676 | Adamts20 | 2.62 | 0.099 | -203 |
| chr15:94373698-94374155 | Irak4 | 2.13 | 0.039 | -163 |
| chr15:94373698-94374155 | Pus7l | 2.13 | 0.039 | 11 |
| chr15:98267872-98268347 | Olfr282 | 2.3 | 0.167 | 209 |
| chr15:98783433-98784106 | Tuba1a | 2.35 | 0.140 | 162 |
| chr15:98859986-98860238 | Tuba1c | 2.4 | 0.046 | -209 |
| chr15:99431575-99432027 | Aqp6 | 2.52 | 0.076 | -29 |
| chr15:99602605-99602851 | Lass5 | 2.48 | 0.122 | 218 |
| chr15:101404056-101404596 | Krt75 | 2.66 | 0.161 | 9 |
| chr15:101754676-101755330 | Krt4 | 2.9 | 0.168 | 163 |
| chr15:101834572-101835563 | Krt8 | 2.75 | 0.124 | -294 |
| chr16:4420128-4420365 | Adcy9 | 2.45 | 0.072 | -659 |
| chr16:5203606-5203870 | Nagpa | 2.09 | 0.223 | 367 |
| chr16:13256093-13257030 | Mkl2 | 2.56 | 0.090 | -11 |
| chr16:18127890-18128144 | Rtn4r | 2.59 | 0.189 | 219 |
| chr16:18235482-18235734 | Zdhhc8 | 2.3 | 0.035 | -379 |
| chr16:19883975-19884301 | A930003A15Rik | 2.69 | 0.201 | 229 |
| chr16:20096679-20097456 | Klhl24 | 2.95 | 0.137 | -558 |
| chr16:20620857-20622391 | Camk2n2 | 2.4 | 0.100 | -273 |
| chr16:21204463-21204920 | Ephb3 | 2.48 | 0.137 | -175 |
| chr16:28445815-28447360 | Fgf12 | 2.98 | 0.117 | -1274 |
| chr16:57121734-57121987 | Tomm70a | 2.53 | 0.140 | 34 |
| chr16:57548070-57548524 | Filip1l | 2.72 | 0.139 | -1057 |
| chr16:58522929-58523674 | St3gal6 | 2.57 | 0.094 | 123 |
| chr16:78301238-78302306 | Cxadr | 3.22 | 0.122 | -163 |
| chr16:84774097-84774824 | Jam2 | 3.23 | 0.197 | 93 |
| chr16:85173459-85173702 | App | 2.24 | 0.135 | 371 |
| chr16:87432563-87432816 | Ltn1 | 2.49 | 0.089 | 161 |
| chr16:88290018-88290468 | Grik1 | 2.76 | 0.125 | 260 |
| chr16:90284171-90284728 | Srsf15 | 2.67 | 0.148 | 220 |
| chr16:91597427-91597677 | Tmem50b | 2.26 | 0.144 | 373 |
| chr16:93603866-93604726 | Setd4 | 2.53 | 0.074 | -236 |
| chr16:96413380-96414663 | Lca5l | 3.43 | 0.171 | -157 |
| chr17:6106948-6107183 | Tulp4 | 2.05 | 0.076 | 236 |
| chr17:12777134-12777368 | Slc22a2 | 2.5 | 0.108 | 197 |
| chr17:12934372-12934624 | Airn | 2.22 | 0.091 | 322 |
| chr17:13897690-13898019 | Tcte2 | 2.28 | 0.077 | 546 |
| chr17:13897690-13898019 | Mllt4 | 2.28 | 0.077 | 307 |
| chr17:15635166-15635413 | Psmb1 | 2.12 | 0.072 | -49 |
| chr17:17761463-17761723 | Lnpep | 2.54 | 0.128 | -140 |
| chr17:24787366-24788333 | Slc9a3r2 | 2.44 | 0.087 | -599 |
| chr17:24872902-24873176 | Sepx1 | 2.17 | 0.041 | -547 |
| chr17:24888663-24889605 | Hs3st6 | 3.21 | 0.140 | -813 |
| chr17:25031638-25031900 | Eme2 | 2.01 | 0.125 | 263 |
| chr17:25031638-25031900 | Mrps34 | 2.01 | 0.125 | -295 |
| chr17:28278171-28278830 | Scube3 | 2.44 | 0.235 | -969 |
| chr17:34256821-34257544 | Brd2 | 2.72 | 0.186 | 145 |
| chr17:34324513-34324808 | Tap1 | 2.04 | 0.050 | 160 |
| chr17:34324513-34324808 | Psmb9 | 2.04 | 0.050 | -385 |
| chr17:34974386-34974836 | Stk19 | 2.64 | 0.064 | -763 |
| chr17:34974386-34974836 | Dom3z | 2.64 | 0.064 | 648 |
| chr17:35226034-35226710 | Bat5 | 2.44 | 0.146 | 137 |
| chr17:35258190-35258656 | Bat4 | 2.13 | 0.071 | -1473 |
| chr17:35258190-35258656 | Bat4 | 2.13 | 0.071 | -17 |
| chr17:35258190-35258656 | Csnk2b | 2.13 | 0.071 | -31 |
| chr17:43153606-43153861 | Tnfrsf21 | 2.13 | 0.122 | 230 |
| chr17:44215805-44216046 | Enpp5 | 2.4 | 0.189 | 131 |
| chr17:44215805-44216046 | Enpp5 | 2.4 | 0.189 | 129 |
| chr17:45709927-45710430 | Hsp90ab1 | 2.63 | 0.105 | 31 |
| chr17:53617883-53618847 | Rab5a | 2.51 | 0.136 | -193 |
| chr17:56176679-56177011 | Sh3gl1 | 2.32 | 0.148 | -846 |
| chr17:56279322-56279635 | Sema6b | 2.54 | 0.171 | 287 |
| chr17:56723325-56723983 | Safb2 | 2.6 | 0.175 | 352 |
| chr17:56723325-56723983 | Safb | 2.6 | 0.175 | -750 |
| chr17:56856062-56857221 | Vmac | 2.81 | 0.077 | 480 |
| chr17:56856062-56857221 | Vmac | 2.81 | 0.077 | -297 |
| chr17:56856062-56857221 | Ndufa11 | 2.81 | 0.077 | -542 |
| chr17:68622837-68623329 | L3mbtl4 | 2.44 | 0.098 | -53 |
| chr17:75404312-75405185 | Ltbp1 | 2.16 | 0.137 | -119 |
| chr17:84586998-84587753 | Zfp36l2 | 2.58 | 0.072 | -88 |
| chr17:88071872-88072114 | Msh2 | 2.01 | 0.129 | 97 |
| chr17:89066917-89068062 | Gtf2a1l | 2.76 | 0.169 | -509 |
| chr18:9958148-9958401 | Thoc1 | 2.49 | 0.111 | 97 |
| chr18:14840924-14841598 | Ss18 | 2.94 | 0.120 | 162 |
| chr18:36356974-36357721 | Nrg2 | 2.36 | 0.129 | -533 |
| chr18:37797880-37799058 | Slc25a2 | 2.77 | 0.123 | -92 |
| chr18:43058676-43059115 | Ppp2r2b | 2.63 | 0.143 | -252 |
| chr18:58369084-58369330 | Fbn2 | 2.31 | 0.078 | 373 |
| chr18:63081501-63081885 | Apcdd1 | 2.2 | 0.118 | -287 |
| chr18:77304044-77304715 | Pias2 | 2.85 | 0.273 | 433 |
| chr18:77304044-77304715 | Pias2 | 2.85 | 0.273 | -38 |
| chr18:77952735-77953409 | 4930465K10Rik | 2.27 | 0.078 | 150 |
| chr18:77952735-77953409 | 8030462N17Rik | 2.27 | 0.078 | -323 |
| chr18:89938483-89938747 | Dok6 | 2.29 | 0.098 | -87 |
| chr18:74938979-74939229 | Acaa2 | 2.48 | 0.198 | 239 |
| chr19:6235343-6235598 | Ppp2r5b | 2.62 | 0.216 | 369 |
| chr19:6384008-6384259 | Pygm | 2.25 | 0.076 | -294 |
| chr19:6996730-6996985 | Esrra | 2.41 | 0.043 | -559 |
| chr19:7049641-7049905 | Ppp1r14b | 2.22 | 0.163 | 236 |
| chr19:8910705-8911276 | Bscl2 | 2.23 | 0.109 | -965 |
| chr19:23215531-23215972 | Klf9 | 2.25 | 0.089 | 36 |
| chr19:29326117-29326368 | Jak2 | 2.44 | 0.120 | -74 |
| chr19:38598377-38599040 | Plce1 | 2.94 | 0.208 | 22 |
| chr19:40588173-40588409 | Sorbs1 | 2.1 | 0.046 | -65 |
| chr19:40588173-40588409 | Sorbs1 | 2.1 | 0.046 | 11 |
| chr19:42853980-42854504 | Hps1 | 2.47 | 0.093 | 224 |
| chr19:45309847-45310106 | Lbx1 | 2.16 | 0.134 | -250 |
| chr19:60302102-60302562 | D19Ertd737e | 2.78 | 0.093 | 268 |
| chr19:60937045-60937348 | Sfxn4 | 2.47 | 0.128 | 136 |
| chrX:17722855-17723097 | Dusp21 | 2.52 | 0.225 | -19 |
| chrX:33651973-33652230 | Il13ra1 | 2.61 | 0.174 | -31 |
| chrX:43681723-43681983 | Actrt1 | 2.64 | 0.124 | -330 |
| chrX:49153977-49154643 | Usp26 | 2.47 | 0.147 | 100 |
| chrX:50341360-50341608 | Hprt | 2.49 | 0.085 | 230 |
| chrX:54340963-54341220 | Vgll1 | 2.38 | 0.063 | -190 |
| chrX:64947225-64947899 | Gm1140 | 2.89 | 0.093 | -1310 |
| chrX:64947225-64947899 | Gm14692 | 2.89 | 0.093 | -825 |
| chrX:64947225-64947899 | Gm1140 | 2.89 | 0.093 | -825 |
| chrX:64947225-64947899 | Gm14692 | 2.89 | 0.093 | -1310 |
| chrX:70363175-70363621 | Xlr5a | 2.38 | 0.062 | -357 |
| chrX:71166992-71167446 | Naa10 | 2.81 | 0.080 | 64 |
| chrX:72660667-72662462 | Brcc3 | 2.35 | 0.103 | -401 |
| chrX:72660667-72662462 | Mtcp1 | 2.35 | 0.103 | 317 |
| chrX:100129076-100130382 | Gm5126 | 2.87 | 0.174 | -167 |
| chrX:103415913-103417461 | Taf9b | 2.98 | 0.140 | -190 |
| chrX:103415913-103417461 | Taf9b | 2.98 | 0.140 | -1506 |
| chrX:131907700-131908203 | Gm5128 | 2.49 | 0.099 | 129 |
| chrX:132738976-132739492 | Tceal5 | 2.65 | 0.109 | -844 |
| chrX:133572562-133573378 | Mcart6 | 2.32 | 0.128 | -129 |
| chrX:133584206-133584456 | Fam199x | 2.17 | 0.233 | 199 |
| chrX:136144976-136145635 | Rnf128 | 2.33 | 0.113 | 147 |
| chrX:141122164-141122832 | Trpc5 | 2.56 | 0.177 | 225 |
| chrX:141122164-141122832 | Zcchc16 | 2.56 | 0.177 | -951 |
| chrX:149204302-149204756 | Shroom2 | 2.44 | 0.050 | -525 |
| chrX:151773185-151773753 | Prdx4 | 2.31 | 0.127 | -472 |
| chrX:162948323-162948572 | Tceanc | 2.4 | 0.053 | -37 |
| chrX:166416768-166418062 | G530011O06Rik | 3.65 | 0.238 | -566 |
| chrY:1919405-1919659 | Sry | 2.19 | 0.164 | 36 |
| 383 hypomethylated peaks | | | | |
| chr1:15302496-15302750 | Kcnb2 | 2.24 | 0.061 | 91 |
| chr1:15795078-15796030 | Terf1 | 2.53 | 0.156 | -184 |
| chr1:33895478-33896126 | Gm15455 | 2.06 | 0.109 | -43 |
| chr1:57433466-57435498 | 1700066M21Rik | 2.77 | 0.086 | 19 |
| chr1:74323875-74324150 | Gpbar1 | 2.32 | 0.131 | -1160 |
| chr1:75313103-75313551 | Dnpep | 2.32 | 0.115 | 265 |
| chr1:107559975-107560838 | 2310035C23Rik | 2.43 | 0.073 | -30 |
| chr1:107559975-107560838 | Pign | 2.43 | 0.073 | -153 |
| chr1:133141257-133142120 | Rassf5 | 3.07 | 0.113 | 66 |
| chr1:138521208-138521460 | Zfp281 | 2.22 | 0.106 | -143 |
| chr1:169618404-169619259 | Lmx1a | 2.68 | 0.090 | -856 |
| chr1:172573985-172574632 | Olfml2b | 2.32 | 0.049 | -353 |
| chr1:173291457-173291924 | Klhdc9 | 2.46 | 0.065 | -761 |
| chr1:184054081-184055036 | Srp9 | 2.53 | 0.058 | -308 |
| chr1:192802803-192803460 | Vash2 | 2.15 | 0.063 | -254 |
| chr1:192802803-192803460 | Vash2 | 2.15 | 0.063 | 43 |
| chr1:194978857-194979789 | Irf6 | 2.86 | 0.148 | 18 |
| chr2:24935157-24936224 | Entpd8 | 3.07 | 0.169 | -151 |
| chr2:25477277-25477731 | Tmem141 | 2.06 | 0.076 | 21 |
| chr2:25477277-25477731 | Tmem141 | 2.06 | 0.076 | 18 |
| chr2:26207556-26208051 | Dnlz | 2.48 | 0.083 | -173 |
| chr2:26864969-26865653 | 5930434B04Rik | 2.68 | 0.166 | -174 |
| chr2:31494465-31495326 | Prdm12 | 2.97 | 0.136 | -660 |
| chr2:37631304-37632017 | Crb2 | 2.88 | 0.110 | -107 |
| chr2:73109922-73110382 | Sp9 | 2.47 | 0.142 | 170 |
| chr2:84876758-84877910 | Ssrp1 | 2.68 | 0.104 | -23 |
| chr2:84876758-84877910 | P2rx3 | 2.68 | 0.104 | -1343 |
| chr2:84876758-84877910 | Ssrp1 | 2.68 | 0.104 | -273 |
| chr2:94246682-94246969 | 2810002D19Rik | 2.78 | 0.061 | -37 |
| chr2:94246682-94246969 | Ttc17 | 2.78 | 0.061 | 20 |
| chr2:103143308-103143758 | Ehf | 2.26 | 0.164 | -180 |
| chr2:109533558-109533807 | Bdnf | 2.02 | 0.028 | -36 |
| chr2:112294946-112295396 | 2900064A13Rik | 2.34 | 0.063 | -10 |
| chr2:115901538-115902030 | 2810405F15Rik | 2.31 | 0.134 | 48 |
| chr2:127482966-127483430 | Mal | 2.23 | 0.148 | -767 |
| chr2:129837946-129838412 | Tgm3 | 2.51 | 0.214 | 70 |
| chr2:145526344-145527396 | BC039771 | 2.23 | 0.114 | -60 |
| chr2:146838655-146839101 | Xrn2 | 3.1 | 0.169 | 83 |
| chr2:148220355-148221428 | Sstr4 | 3.18 | 0.113 | -220 |
| chr2:154233321-154233994 | Snta1 | 2.03 | 0.090 | 162 |
| chr2:156137224-156138094 | 4921517L17Rik | 2.55 | 0.134 | -549 |
| chr2:156137224-156138094 | Scand1 | 2.55 | 0.134 | 781 |
| chr2:160737566-160738637 | Emilin3 | 2.98 | 0.107 | -37 |
| chr2:168055338-168057145 | Dpm1 | 3.51 | 0.173 | -362 |
| chr2:168055338-168057145 | Mocs3 | 3.51 | 0.173 | 120 |
| chr3:7366153-7367032 | Pkia | 3.2 | 0.170 | -10 |
| chr3:33742963-33743216 | Ccdc39 | 2.47 | 0.074 | 142 |
| chr3:66786607-66786860 | Shox2 | 2.17 | 0.078 | -1040 |
| chr3:67177671-67178140 | Mlf1 | 2.28 | 0.083 | -112 |
| chr3:81949305-81949554 | Gucy1a3 | 2.54 | 0.119 | 369 |
| chr3:90330153-90330634 | S100a14 | 2.16 | 0.053 | -376 |
| chr3:90403448-90404508 | S100a3 | 2.66 | 0.124 | -158 |
| chr3:100292823-100293535 | Fam46c | 2.5 | 0.192 | -64 |
| chr3:100773087-100773751 | Ttf2 | 2.69 | 0.087 | 167 |
| chr3:108882853-108883322 | 4930443G12Rik | 2.22 | 0.100 | -328 |
| chr3:110053634-110053894 | Prmt6 | 2.14 | 0.144 | 152 |
| chr3:126300093-126300353 | Camk2d | 2.74 | 0.244 | 333 |
| chr3:142363605-142364055 | Ccbl2 | 2.56 | 0.101 | -213 |
| chr4:24825267-24825515 | Ndufaf4 | 3 | 0.187 | 162 |
| chr4:33010629-33010889 | Ankrd6 | 2.12 | 0.026 | -279 |
| chr4:35099322-35099603 | Ifnk | 2.23 | 0.190 | 159 |
| chr4:42248200-42248445 | Ccl19 | 2.31 | 0.038 | -180 |
| chr4:43394089-43395157 | Rusc2 | 3.39 | 0.134 | -230 |
| chr4:70196093-70197148 | Megf9 | 3.47 | 0.138 | -658 |
| chr4:88525818-88526275 | Ifne | 2.48 | 0.079 | 58 |
| chr4:102258863-102259371 | Pde4b | 2.52 | 0.145 | -1045 |
| chr4:106350796-106351451 | Ttc4 | 3.35 | 0.182 | 167 |
| chr4:106350796-106351451 | Ttc4 | 3.35 | 0.182 | 425 |
| chr4:107562150-107562809 | 0610037L13Rik | 3.07 | 0.140 | -23 |
| chr4:120338821-120339284 | Cited4 | 2.33 | 0.061 | -114 |
| chr4:123020407-123021384 | Bmp8a | 2.75 | 0.187 | -849 |
| chr4:129317552-129317800 | Txlna | 2.36 | 0.072 | 208 |
| chr4:137548136-137548405 | Eif4g3 | 2.43 | 0.092 | -1113 |
| chr4:137951878-137952740 | Fam43b | 2.63 | 0.138 | 64 |
| chr4:137990169-137990867 | Mul1 | 2.81 | 0.155 | -68 |
| chr4:139388384-139388636 | Pax7 | 2.15 | 0.095 | 373 |
| chr4:149329243-149330309 | Spsb1 | 2.51 | 0.101 | -661 |
| chr4:151307663-151308117 | Dnajc11 | 2.04 | 0.136 | 62 |
| chr4:151363423-151363871 | Thap3 | 2.25 | 0.045 | -552 |
| chr4:151670883-151671548 | Icmt | 2.36 | 0.117 | -243 |
| chr4:151698273-151699643 | Rpl22 | 2.32 | 0.108 | -1028 |
| chr4:153673714-153673970 | Arhgef16 | 2.34 | 0.065 | 162 |
| chr5:8422881-8423437 | Dbf4 | 2.26 | 0.046 | -443 |
| chr5:8422881-8423437 | Slc25a40 | 2.26 | 0.046 | 310 |
| chr5:15439214-15439677 | Cacna2d1 | 2.6 | 0.121 | -1062 |
| chr5:29704436-29705104 | Lmbr1 | 2.82 | 0.214 | 160 |
| chr5:34063497-34064567 | Fgfr3 | 2.23 | 0.050 | -922 |
| chr5:34063497-34064567 | Fgfr3 | 2.23 | 0.050 | -376 |
| chr5:34063497-34064567 | Fgfr3 | 2.23 | 0.050 | -340 |
| chr5:39076720-39077180 | Zfp518b | 2.82 | 0.211 | -885 |
| chr5:43623511-43624018 | Cpeb2 | 2.7 | 0.178 | -936 |
| chr5:45840969-45841847 | Qdpr | 2.69 | 0.102 | 60 |
| chr5:65781458-65782121 | Lias | 2.6 | 0.187 | -945 |
| chr5:88128380-88128845 | Csn2 | 2.05 | 0.063 | -166 |
| chr5:88408905-88409416 | 4931407G18Rik | 2.52 | 0.088 | 685 |
| chr5:92706913-92707581 | Naaa | 2.18 | 0.065 | -40 |
| chr5:99465805-99466059 | Prkg2 | 2.24 | 0.152 | 166 |
| chr5:108741465-108742384 | Pigg | 2.68 | 0.113 | -18 |
| chr5:109058734-109059834 | Tmem175 | 3.15 | 0.129 | 456 |
| chr5:109058734-109059834 | Gak | 3.15 | 0.129 | -526 |
| chr5:111199451-111200123 | Ep400 | 2.46 | 0.117 | -51 |
| chr5:118426921-118427177 | Fbxo21 | 2.81 | 0.152 | 271 |
| chr5:121228692-121228937 | Oas3 | 2.67 | 0.190 | -1146 |
| chr5:123271230-123271478 | Anapc5 | 2.13 | 0.102 | -6 |
| chr5:123582139-123583015 | Rhof | 2.82 | 0.108 | 61 |
| chr5:124777960-124778215 | Mphosph9 | 3.15 | 0.075 | 372 |
| chr5:124777960-124778215 | 2810006K23Rik | 3.15 | 0.075 | -9 |
| chr5:135582743-135583198 | Mlxipl | 2.48 | 0.136 | 188 |
| chr5:136219546-136220007 | Tmem120a | 2.92 | 0.121 | 265 |
| chr5:137504988-137505232 | Vgf | 2.58 | 0.138 | -1054 |
| chr5:138721312-138721561 | Stag3 | 2.11 | 0.067 | -299 |
| chr5:138721312-138721561 | Gpc2 | 2.11 | 0.067 | -271 |
| chr5:140288706-140288955 | Tmem184a | 2.68 | 0.141 | 366 |
| chr5:143177822-143178077 | Slc29a4 | 2.1 | 0.139 | -104 |
| chr5:147775504-147776169 | Mtif3 | 2.22 | 0.089 | -486 |
| chr5:149995687-149996142 | 5730422E09Rik | 2.38 | 0.059 | -275 |
| chr5:149995687-149996142 | Uspl1 | 2.38 | 0.059 | -220 |
| chr6:3714138-3714590 | Calcr | 2.52 | 0.077 | 349 |
| chr6:3714138-3714590 | Calcr | 2.52 | 0.077 | -741 |
| chr6:42694251-42694500 | Olfr453 | 2.73 | 0.249 | 338 |
| chr6:47997667-47998025 | Zfp777 | 2.49 | 0.148 | 267 |
| chr6:52114537-52114842 | Hoxa2 | 2.11 | 0.088 | 140 |
| chr6:71390454-71391163 | Rmnd5a | 2.32 | 0.147 | -177 |
| chr6:72312096-72312751 | Rnf181 | 2.02 | 0.073 | -48 |
| chr6:72849846-72850773 | Kcmf1 | 2.2 | 0.127 | -336 |
| chr6:73171559-73172016 | Dnahc6 | 2.06 | 0.100 | -162 |
| chr6:83105375-83107151 | 1700003E16Rik | 2.67 | 0.096 | -134 |
| chr6:83105375-83107151 | Wdr54 | 2.67 | 0.096 | 110 |
| chr6:88171587-88172461 | Dnajb8 | 2.3 | 0.053 | -237 |
| chr6:113256446-113257581 | Brpf1 | 2.59 | 0.103 | -176 |
| chr6:113276174-113277049 | Ogg1 | 2.31 | 0.058 | -357 |
| chr6:113481399-113481661 | Tmem111 | 2.32 | 0.129 | 102 |
| chr6:113481399-113481661 | Fancd2 | 2.32 | 0.129 | -145 |
| chr6:113646457-113646728 | Tatdn2 | 2.35 | 0.112 | -899 |
| chr6:116624644-116624894 | Rassf4 | 2.44 | 0.059 | -915 |
| chr6:120615139-120615873 | Cecr2 | 2.55 | 0.109 | -932 |
| chr6:122692848-122693508 | Slc2a3 | 2.27 | 0.040 | -415 |
| chr6:124662223-124662491 | Emg1 | 2.51 | 0.053 | -161 |
| chr6:124662223-124662491 | Phb2 | 2.51 | 0.053 | 51 |
| chr6:126799537-126799791 | Ndufa9 | 2.29 | 0.100 | -502 |
| chr6:127402649-127404028 | Parp11 | 2.52 | 0.070 | -401 |
| chr6:134847821-134848384 | Gpr19 | 2.32 | 0.101 | -159 |
| chr6:134847821-134848384 | Gpr19 | 2.32 | 0.101 | -269 |
| chr6:134847821-134848384 | 2810454H06Rik | 2.32 | 0.101 | 124 |
| chr6:136776071-136776313 | BC049715 | 2.15 | 0.208 | -1171 |
| chr6:136776071-136776313 | Wbp11 | 2.15 | 0.208 | 545 |
| chr6:149089892-149090150 | 4833442J19Rik | 2.43 | 0.141 | -97 |
| chr7:4289345-4289801 | Ncr1 | 2.87 | 0.156 | 248 |
| chr7:6335783-6336294 | Zfp28 | 2.53 | 0.081 | 11 |
| chr7:16806856-16807646 | Dhx34 | 2.94 | 0.186 | 130 |
| chr7:17578894-17579182 | Ccdc8 | 2.44 | 0.154 | -898 |
| chr7:20162554-20162799 | Zfp296 | 2.16 | 0.054 | 41 |
| chr7:25861831-25862090 | Zfp574 | 2.47 | 0.041 | -303 |
| chr7:25861831-25862090 | Zfp574 | 2.47 | 0.041 | -262 |
| chr7:31348204-31348449 | Psenen | 2.45 | 0.052 | -123 |
| chr7:31348204-31348449 | U2af1l4 | 2.45 | 0.052 | -31 |
| chr7:52466762-52467835 | Dkkl1 | 2.99 | 0.147 | -45 |
| chr7:53069201-53070131 | Cyth2 | 2.77 | 0.129 | 20 |
| chr7:53493364-53493625 | Ush1c | 2.53 | 0.164 | 365 |
| chr7:54050858-54051311 | Gtf2h1 | 2.56 | 0.095 | -378 |
| chr7:54050858-54051311 | Hps5 | 2.56 | 0.095 | 166 |
| chr7:73224686-73224926 | H47 | 2.59 | 0.150 | 272 |
| chr7:86536495-86537590 | Fanci | 2.65 | 0.176 | -180 |
| chr7:88045671-88045916 | Wdr73 | 2.46 | 0.100 | 361 |
| chr7:91234628-91234871 | 9930013L23Rik | 2.04 | 0.076 | 265 |
| chr7:99785157-99785417 | Ankrd42 | 2.6 | 0.150 | 365 |
| chr7:107269517-107269766 | Pold3 | 2.8 | 0.111 | 368 |
| chr7:109248654-109248918 | Art5 | 2.32 | 0.058 | -43 |
| chr7:118226260-118227541 | Eif4g2 | 2.97 | 0.115 | -356 |
| chr7:126668217-126668828 | Acsm5 | 2.98 | 0.173 | -1255 |
| chr7:126668217-126668828 | Pdilt | 2.98 | 0.173 | -1526 |
| chr7:134027990-134028241 | Taok2 | 2.2 | 0.167 | 365 |
| chr7:134234010-134234507 | AI467606 | 2.9 | 0.157 | -690 |
| chr7:142729628-142729882 | Ptpre | 2.43 | 0.105 | 249 |
| chr7:148599757-148600914 | Pddc1 | 2.82 | 0.082 | -311 |
| chr7:149620038-149620289 | Syt8 | 2.51 | 0.081 | -717 |
| chr7:150687941-150688395 | Phlda2 | 2.32 | 0.202 | 261 |
| chr8:3720261-3720800 | Clec4g | 2.17 | 0.146 | 120 |
| chr8:8660903-8661144 | Efnb2 | 2.14 | 0.062 | -250 |
| chr8:14094621-14095086 | Dlgap2 | 2.43 | 0.159 | -1020 |
| chr8:18741354-18741611 | Angpt2 | 2.41 | 0.055 | 68 |
| chr8:23235045-23235495 | Nek5 | 2.53 | 0.085 | 255 |
| chr8:35171076-35171740 | Dctn6 | 2.84 | 0.170 | 157 |
| chr8:35217123-35217583 | Tmem66 | 2.01 | 0.081 | -263 |
| chr8:36558708-36559213 | Eri1 | 2.41 | 0.069 | -373 |
| chr8:63471415-63472359 | Nek1 | 2.74 | 0.160 | -129 |
| chr8:74405244-74405527 | Zfp709 | 2.2 | 0.123 | -580 |
| chr8:83017565-83018034 | Gypa | 2.75 | 0.069 | -143 |
| chr8:96337368-96338553 | 4930488L21Rik | 3.01 | 0.168 | -505 |
| chr8:97604216-97604895 | Katnb1 | 2.18 | 0.043 | -544 |
| chr8:107799672-107800621 | Nol3 | 2.48 | 0.107 | -199 |
| chr8:109394020-109394476 | Has3 | 2.74 | 0.154 | 107 |
| chr8:121960287-121960531 | Osgin1 | 2.46 | 0.089 | -652 |
| chr8:124430433-124431107 | BC048644 | 2.6 | 0.195 | -962 |
| chr8:124430433-124431107 | Slc7a5 | 2.6 | 0.195 | 816 |
| chr9:7571019-7571898 | Mmp27 | 3.27 | 0.211 | 1 |
| chr9:20835200-20835527 | Icam5 | 2.89 | 0.248 | -1117 |
| chr9:36604155-36604828 | Ei24 | 2.84 | 0.144 | 161 |
| chr9:44306361-44306622 | Bcl9l | 2.5 | 0.056 | -726 |
| chr9:50665126-50665366 | Ppp2r1b | 2.6 | 0.147 | 207 |
| chr9:64799244-64799951 | Slc24a1 | 2.58 | 0.115 | -183 |
| chr9:65246064-65246308 | Rasl12 | 2.18 | 0.059 | -108 |
| chr9:65907793-65908464 | Ppib | 2.65 | 0.158 | 153 |
| chr9:72891607-72892060 | Rab27a | 2.6 | 0.183 | -837 |
| chr9:78038802-78039133 | Gsta4 | 2.3 | 0.123 | -804 |
| chr9:96019032-96020166 | Gk5 | 2.38 | 0.072 | -248 |
| chr9:96918275-96918524 | Spsb4 | 2.19 | 0.081 | 374 |
| chr9:106269868-106270334 | Dusp7 | 2.32 | 0.084 | -861 |
| chr9:106331717-106332168 | Rpl29 | 2.38 | 0.091 | 73 |
| chr9:107445046-107445295 | Cyb561d2 | 2.56 | 0.146 | -974 |
| chr9:114639689-114640377 | Cmtm6 | 2.16 | 0.136 | -287 |
| chr10:5916803-5917061 | Gm5512 | 2.38 | 0.044 | -442 |
| chr10:20067342-20067606 | Fam54a | 2.19 | 0.109 | -150 |
| chr10:24589217-24590094 | Med23 | 3.38 | 0.137 | -135 |
| chr10:61110154-61110856 | Ppa1 | 2.67 | 0.127 | -863 |
| chr10:75700344-75701835 | Prmt2 | 2.99 | 0.110 | -479 |
| chr10:77531813-77532476 | Icosl | 2.32 | 0.105 | 32 |
| chr10:78560166-78560821 | 2610008E11Rik | 2.35 | 0.175 | -148 |
| chr10:81089583-81090058 | Sirt6 | 2.46 | 0.131 | 246 |
| chr10:81089583-81090058 | Ankrd24 | 2.46 | 0.131 | -1463 |
| chr10:81089583-81090058 | Sirt6 | 2.46 | 0.131 | 532 |
| chr10:82153367-82154030 | Glt8d2 | 2.64 | 0.098 | -303 |
| chr10:93293003-93294205 | Usp44 | 2.63 | 0.093 | -695 |
| chr10:127189605-127190015 | Gpr182 | 2.41 | 0.176 | -956 |
| chr11:3549037-3549962 | Morc2a | 2.66 | 0.121 | 3 |
| chr11:3549037-3549962 | Tug1 | 2.66 | 0.121 | -688 |
| chr11:4135702-4137001 | Osm | 2.26 | 0.115 | -435 |
| chr11:20232221-20232481 | Slc1a4 | 2.71 | 0.250 | 365 |
| chr11:35793505-35794036 | Wwc1 | 2.44 | 0.115 | -179 |
| chr11:40547017-40547470 | Nudcd2 | 2.2 | 0.073 | 100 |
| chr11:40547017-40547470 | Hmmr | 2.2 | 0.073 | -304 |
| chr11:50191195-50191429 | Hnrnph1 | 2.25 | 0.057 | 92 |
| chr11:53246281-53247268 | Gdf9 | 2.68 | 0.107 | -12 |
| chr11:64792342-64792603 | Elac2 | 2.23 | 0.080 | -63 |
| chr11:67584159-67584405 | Glp2r | 2.73 | 0.195 | 373 |
| chr11:69419192-69419706 | Atp1b2 | 2.13 | 0.071 | 13 |
| chr11:70513497-70514782 | Inca1 | 2.74 | 0.111 | -482 |
| chr11:70513497-70514782 | Kif1c | 2.74 | 0.111 | 90 |
| chr11:83755190-83756063 | Ddx52 | 2.6 | 0.183 | 35 |
| chr11:84632530-84633181 | Mrm1 | 2.43 | 0.093 | 161 |
| chr11:86887210-86887878 | Gdpd1 | 2.54 | 0.118 | 95 |
| chr11:96163539-96165269 | Hoxb5 | 3.06 | 0.108 | -421 |
| chr11:96839532-96840202 | Sp2 | 2.58 | 0.135 | -865 |
| chr11:97848218-97848463 | Plxdc1 | 2.22 | 0.070 | -580 |
| chr11:100006945-100008214 | Krt19 | 2.61 | 0.106 | -346 |
| chr11:100948186-100948647 | Mlx | 2.52 | 0.089 | -186 |
| chr11:100980354-100981274 | Tubg1 | 2.86 | 0.068 | -630 |
| chr11:100980354-100981274 | Fam134c | 2.86 | 0.068 | 343 |
| chr11:100980354-100981274 | Fam134c | 2.86 | 0.068 | 324 |
| chr11:105898365-105898609 | Dcaf7 | 2.31 | 0.083 | 302 |
| chr11:106133787-106134871 | Smarcd2 | 2.4 | 0.103 | -43 |
| chr11:119903221-119903469 | Aatk | 2.31 | 0.045 | -250 |
| chr12:33831254-33832835 | F730043M19Rik | 3.1 | 0.206 | 406 |
| chr12:33831254-33832835 | Atxn7l1 | 3.1 | 0.206 | -505 |
| chr12:45310775-45311435 | Dnajb9 | 2.36 | 0.204 | -50 |
| chr12:56181250-56181713 | Srp54a | 2.49 | 0.198 | 0 |
| chr12:56181250-56181713 | 2700097O09Rik | 2.49 | 0.198 | -384 |
| chr12:57637610-57638081 | Nkx2-1 | 2.9 | 0.071 | 49 |
| chr12:60320474-60321402 | Fbxo33 | 2.78 | 0.081 | -468 |
| chr12:77425911-77426174 | Akap5 | 2.6 | 0.114 | 165 |
| chr12:80190364-80191062 | Pigh | 2.31 | 0.079 | -56 |
| chr12:88067127-88067365 | Angel1 | 2.25 | 0.071 | 164 |
| chr12:103677676-103677923 | Lgmn | 2.53 | 0.066 | 107 |
| chr12:113383755-113384689 | Kif26a | 2.74 | 0.162 | -196 |
| chr13:19487401-19487860 | Stard3nl | 2.13 | 0.155 | -9 |
| chr13:21903647-21903907 | Gm11275 | 2.47 | 0.129 | 163 |
| chr13:21903647-21903907 | Hist1h4m | 2.47 | 0.129 | 125 |
| chr13:43880145-43880601 | Cd83 | 2.4 | 0.066 | -102 |
| chr13:59871002-59871870 | Etohd2 | 2.68 | 0.083 | 110 |
| chr13:59871002-59871870 | Isca1 | 2.68 | 0.083 | -286 |
| chr13:107736883-107737602 | Dimt1 | 2.84 | 0.080 | 34 |
| chr13:113824702-113825392 | Mir449c | 2.29 | 0.047 | -1143 |
| chr14:18493211-18493653 | Thrb | 2.35 | 0.071 | -41 |
| chr14:21365044-21365797 | Ppp3cb | 2.29 | 0.144 | 374 |
| chr14:21365044-21365797 | 1810062O18Rik | 2.29 | 0.144 | -93 |
| chr14:33134845-33135504 | Ogdhl | 2.57 | 0.090 | -29 |
| chr14:34029864-34030319 | Arhgap22 | 2.78 | 0.282 | 83 |
| chr14:56159321-56159573 | Pck2 | 2.66 | 0.180 | 345 |
| chr14:56518573-56519244 | Sdr39u1 | 3.07 | 0.132 | 160 |
| chr14:57190186-57190448 | Cenpj | 2.49 | 0.137 | 366 |
| chr14:58189907-58190156 | N6amt2 | 2.77 | 0.161 | 374 |
| chr14:60059740-60059979 | Cab39l | 2.5 | 0.124 | 42 |
| chr14:60059740-60059979 | Setdb2 | 2.5 | 0.124 | -145 |
| chr14:61979226-61979470 | Ebpl | 2.45 | 0.059 | -66 |
| chr14:69789922-69791003 | Nkx2-6 | 3.07 | 0.179 | 387 |
| chr14:70843363-70844344 | Mir320 | 3.67 | 0.181 | 537 |
| chr14:70843363-70844344 | Polr3d | 3.67 | 0.181 | -819 |
| chr14:70843363-70844344 | Polr3d | 3.67 | 0.181 | -575 |
| chr14:70857201-70857676 | Phyhip | 2.57 | 0.129 | 115 |
| chr14:73725540-73725827 | Rb1 | 2.49 | 0.114 | -85 |
| chr14:118665289-118666766 | Gm9376 | 2.56 | 0.163 | -351 |
| chr15:39028542-39028787 | Rims2 | 2.49 | 0.110 | -1212 |
| chr15:58766080-58766581 | Tatdn1 | 3.1 | 0.187 | -1045 |
| chr15:73253122-73253912 | Ptk2 | 2.47 | 0.100 | 104 |
| chr15:75911418-75911679 | Puf60 | 2.04 | 0.068 | -172 |
| chr15:75911418-75911679 | Puf60 | 2.04 | 0.068 | -248 |
| chr15:76123577-76124866 | Gm10345 | 2.69 | 0.112 | -641 |
| chr15:77136987-77137281 | Rbfox2 | 2.29 | 0.117 | 349 |
| chr15:79273025-79273312 | Csnk1e | 2.38 | 0.136 | -681 |
| chr15:81295528-81295868 | Rbx1 | 2.7 | 0.156 | -1047 |
| chr15:83002344-83003223 | Cyb5r3 | 2.39 | 0.064 | -145 |
| chr15:98918367-98918623 | C1ql4 | 2.36 | 0.090 | -336 |
| chr15:99482013-99482284 | Racgap1 | 2.53 | 0.108 | -96 |
| chr15:100946352-100946597 | Ankrd33 | 2.29 | 0.155 | 289 |
| chr15:102019657-102020659 | Csad | 3.36 | 0.263 | -684 |
| chr16:11254891-11255577 | Gspt1 | 2.73 | 0.139 | -816 |
| chr16:11254891-11255577 | Mir1945 | 2.73 | 0.139 | -696 |
| chr16:13109181-13109415 | Ercc4 | 2.34 | 0.182 | -530 |
| chr16:13667778-13668233 | Parn | 2.39 | 0.099 | 257 |
| chr16:17530866-17531115 | Thap7 | 2.42 | 0.088 | 154 |
| chr16:29544666-29544908 | Atp13a4 | 2.52 | 0.126 | 163 |
| chr16:32418894-32420108 | Tctex1d2 | 2.95 | 0.136 | -286 |
| chr16:38346541-38347430 | Cox17 | 2.54 | 0.148 | -98 |
| chr16:72662752-72663254 | Robo1 | 2.07 | 0.153 | -390 |
| chr17:6079693-6080137 | Serac1 | 2.05 | 0.040 | -176 |
| chr17:6079693-6080137 | Gtf2h5 | 2.05 | 0.040 | 88 |
| chr17:15636440-15637343 | Psmb1 | 3.42 | 0.261 | -1651 |
| chr17:15636440-15637343 | Tbp | 3.42 | 0.261 | 40 |
| chr17:20481089-20481558 | Vmn2r107 | 2.32 | 0.127 | -1064 |
| chr17:25073423-25073888 | Mapk8ip3 | 2.86 | 0.160 | 266 |
| chr17:25252352-25253421 | Telo2 | 3.78 | 0.162 | -36 |
| chr17:25252352-25253421 | Telo2 | 3.78 | 0.162 | 25 |
| chr17:26231930-26232790 | Nme4 | 2.31 | 0.083 | 55 |
| chr17:28487865-28488396 | Tead3 | 2.99 | 0.086 | -380 |
| chr17:28487865-28488396 | Tead3 | 2.99 | 0.086 | -585 |
| chr17:35561147-35562216 | H2-Q6 | 3.34 | 0.116 | -139 |
| chr17:35575903-35576152 | H2-Q7 | 2.16 | 0.112 | -71 |
| chr17:35805116-35805574 | Vars2 | 2.6 | 0.232 | -808 |
| chr17:43526332-43526732 | Gpr116 | 2.07 | 0.179 | 118 |
| chr17:64681315-64681570 | Pja2 | 2.11 | 0.050 | -219 |
| chr17:71947126-71947785 | Trmt61b | 2.71 | 0.118 | 645 |
| chr17:71947126-71947785 | Trmt61b | 2.71 | 0.118 | 165 |
| chr17:79336106-79336582 | Cebpz | 2.9 | 0.164 | 66 |
| chr17:79336106-79336582 | 2410091C18Rik | 2.9 | 0.164 | -130 |
| chr17:79419981-79420309 | Prkd3 | 2.37 | 0.074 | 11 |
| chr17:79754999-79755259 | Cdc42ep3 | 2.27 | 0.060 | -698 |
| chr17:80963738-80963998 | Cdkl4 | 2.65 | 0.123 | -694 |
| chr17:87506058-87506621 | Socs5 | 2.65 | 0.075 | -678 |
| chr18:9212169-9212418 | Fzd8 | 2.4 | 0.089 | -559 |
| chr18:15309471-15309960 | Kctd1 | 2.93 | 0.094 | 239 |
| chr18:34783904-34784833 | Kif20a | 2.9 | 0.098 | -317 |
| chr18:34783904-34784833 | Kif20a | 2.9 | 0.098 | -220 |
| chr18:34783904-34784833 | Brd8 | 2.9 | 0.098 | 95 |
| chr18:34783904-34784833 | Kif20a | 2.9 | 0.098 | 91 |
| chr18:35278011-35278252 | Ctnna1 | 2.08 | 0.072 | -433 |
| chr18:36687660-36688120 | Slc4a9 | 2.62 | 0.102 | 85 |
| chr18:37119232-37120580 | Pcdha5 | 2.86 | 0.112 | -187 |
| chr18:37132278-37133784 | Pcdha7 | 3.22 | 0.286 | -546 |
| chr18:37866767-37867065 | Pcdhga6 | 2.34 | 0.109 | 34 |
| chr18:37911564-37911849 | Pcdhgb7 | 2.17 | 0.144 | 274 |
| chr18:37920815-37921898 | Pcdhgb8 | 2.65 | 0.124 | -97 |
| chr18:38129102-38129753 | Fchsd1 | 2.47 | 0.073 | -42 |
| chr18:44538867-44539321 | Dcp2 | 2.35 | 0.124 | -1059 |
| chr18:46372185-46373102 | Trim36 | 2.78 | 0.080 | -382 |
| chr18:67884186-67884891 | Ptpn2 | 2.24 | 0.064 | -263 |
| chr18:74938979-74939229 | Scarna17 | 2.48 | 0.198 | -995 |
| chr18:77182397-77183440 | Hdhd2 | 2.37 | 0.097 | 65 |
| chr18:77182397-77183440 | Hdhd2 | 2.37 | 0.097 | -237 |
| chr18:90679218-90679948 | Tmx3 | 2.73 | 0.150 | 38 |
| chr19:3575957-3576896 | Ppp6r3 | 2.77 | 0.088 | -677 |
| chr19:4711163-4711417 | Spnb3 | 2.35 | 0.128 | 68 |
| chr19:5459725-5460753 | Fibp | 2.57 | 0.139 | -454 |
| chr19:8967275-8967949 | Ints5 | 2.6 | 0.127 | 136 |
| chr19:10680094-10680348 | Ddb1 | 2.44 | 0.060 | 107 |
| chr19:12502387-12502845 | Pfpl | 2.41 | 0.149 | 222 |
| chr19:17431278-17431537 | Gcnt1 | 2.08 | 0.034 | -250 |
| chr19:29595975-29597054 | C030046E11Rik | 2.25 | 0.054 | -256 |
| chr19:29595975-29597054 | A930007I19Rik | 2.25 | 0.054 | -37 |
| chr19:41970202-41970866 | Rrp12 | 3.03 | 0.223 | 109 |
| chr19:46673231-46673494 | D19Wsu162e | 2.21 | 0.056 | -232 |
| chr19:46781793-46782247 | As3mt | 2.57 | 0.130 | 88 |
| chr19:57435948-57436611 | Fam160b1 | 2.33 | 0.177 | 781 |
| chrX:8540538-8541192 | Gm5634 | 2.61 | 0.124 | -764 |
| chrX:8553906-8554164 | Gm14511 | 2.02 | 0.083 | -350 |
| chrX:13043794-13044678 | Nyx | 3.39 | 0.234 | -561 |
| chrX:20911628-20911880 | Gm6938 | 2.05 | 0.112 | 99 |
| chrX:35179708-35180377 | Rhox6 | 2.29 | 0.121 | -188 |
| chrX:69062152-69062421 | 2610030H06Rik | 2.65 | 0.105 | 169 |
| chrX:98615977-98617139 | Zmym3 | 2.94 | 0.111 | -534 |
| chrX:98615977-98617139 | Zmym3 | 2.94 | 0.111 | -1305 |
| chrX:98615977-98617139 | Zmym3 | 2.94 | 0.111 | -997 |
| chrX:130442733-130443182 | Srpx2 | 2.43 | 0.121 | -6 |
| chrX:160707408-160707733 | Pir | 2.6 | 0.158 | 208 |

Peak Score: The average of -log_10_(P value) from probes within the peak. The scores reflect the probability of positive enrichment (Cut off = 2). Peak M-value: The median of log_2_(MeDIP/Input) from probes within the peak region. The score reflects the hydroxymethylation level of the region. TSS: transcriptional starting site
